# The essential neutral sphingomyelinase is involved in the trafficking of the variant surface glycoprotein in the bloodstream form of *Trypanosoma brucei*

**DOI:** 10.1111/j.1365-2958.2010.07151.x

**Published:** 2010-04-15

**Authors:** Simon A Young, Terry K Smith

**Affiliations:** Biomolecular Science, The North Haugh, The University, St. AndrewsFife Scotland KY16 9ST, UK

## Abstract

Sphingomyelin is the main sphingolipid in *Trypanosoma brucei*, the causative agent of African sleeping sickness. *In vitro* and *in vivo* characterization of the *T. brucei* neutral sphingomyelinase demonstrates that it is directly involved in sphingomyelin catabolism. Gene knockout studies in the bloodstream form of the parasite indicate that the neutral sphingomyelinase is essential for growth and survival, thus highlighting that the *de novo* biosynthesis of ceramide is unable to compensate for the loss of sphingomyelin catabolism. The phenotype of the conditional knockout has given new insights into the highly active endocytic and exocytic pathways in the bloodstream form of *T. brucei.* Hence, the formation of ceramide in the endoplasmic reticulum affects post-Golgi sorting and rate of deposition of newly synthesized GPI-anchored variant surface glycoprotein on the cell surface. This directly influences the corresponding rate of endocytosis, via the recycling endosomes, of pre-existing cell surface variant surface glycoprotein. The trypanosomes use this coupled endocytic and exocytic mechanism to maintain the cell density of its crucial variant surface glycoprotein protective coat. *Tb*nSMase is therefore genetically validated as a drug target against African trypanosomes, and suggests that interfering with the endocytic transport of variant surface glycoprotein is a highly desirable strategy for drug development against African trypanosomasis.

## Introduction

The medically important protozoan kinetoplastid *Trypanosoma brucei*, which cause African sleeping sickness, has a complex life cycle that alternates between an insect vector and a mammalian host (Matthews, 2005). Despite this the phospholipid content found in these life cycle stages are remarkably similar (Patnaik *et al*., 1993; Richmond *et al*., 2010). Phosphatidylcholine (PC) and sphingomyelin (SM) are the two most abundant phospholipid species in eukaryotic cells and accounts for over half of the total phospholipids in *T. brucei* membranes (Patnaik *et al*., 1993).

Sphingolipids, the most common of which is SM, are essential structural components of eukaryotic membranes, and function in diverse cellular processes (Cowart *et al*., 2006; Lahiri and Futerman, 2007). They are abundant in the outer leaflet of the plasma membrane of many cells but are also found in lysosomal membranes and endoplasmic reticulum (ER) and the Golgi, where they play an important role in controlling membrane fluidity (Koynova and Caffery, 1995; van Meer *et al*., 2008).

Eukaryotic SM synthase activity has been observed in the plasma membrane, but is primarily localized in the Golgi, where it transfers the choline–phosphate headgroup from PC to ceramide. Ceramide can be formed either via the *de novo* biosynthesis, which commences by the condensation of serine with palmitoyl-CoA, or via hydrolysis (degradation) of complex sphingolipids normally by sphingomyelinases (SMases) (Futerman and Riezman, 2005).

The diversity of sphingolipid synthesis is a developing area for anti-fungals because yeast synthesize significant amounts of inositol phosphorylceramide (IPC), by the transfer of the inositol-1-phosphate headgroup of phosphatidylinositol (PI) to ceramide, which is in contrast to mammals, which only synthesize SM (Dickson *et al*., 2006). Similarly various sphingolipid species and their biosynthesis in kinetoplastid parasites have also drawn attention due to their potential as novel chemotherapeutic targets (Suzuki *et al*., 2008). *Trypanosoma cruzi* and *Leishmania* both synthesize significant amounts of IPC (Kaneshiro *et al*., 1986; Bertello *et al*., 1995; Uhrig *et al*., 1996; Zhang *et al*., 2007) via orthologues of the yeast AUR1 inositol phosphoceramide synthase (Denny *et al*., 2006). The viability of a *Leishmania major* promastigote null of the serine palmitoyltransferase, the first enzyme of the sphingoid base pathway, suggests that *Leishmania* do not require sphingolipids (Zhang *et al*., 2003). However, it was subsequently discovered that they use a sphingosine lyase to generate ethanolamine (Zhang *et al*., 2007).

Procyclic *T. brucei* contain both SM and IPC (Güther *et al*., 2006; Fridberg *et al*., 2008), while bloodstream stage parasites contain no IPC only synthesizing SM and minor amounts of ethanolamine phosphorylceramide (EPC) (Bagnat *et al*., 2000; Sutterwala *et al*., 2008). Recently, it has been discovered that mammalian stumpy form *T. brucei* upregulate a mitochondrial SM synthase (SLS1, Tb09.211.1030) (Kabani *et al*., 2009), responsible for making IPC (Mina *et al*., 2009), and this is paralleled by formation of IPC as identified by electrospray mass spectrometry (ES-MS) (Kabani *et al*., 2009).

Together, these observations clearly indicate that sphingolipid synthesis in African trypanosomes is developmentally regulated. RNAi silencing of the *TbSLS* locus (four tandem genes) leads to growth arrest, suggesting synthesis of some or all sphingolipids are essential in trypanosomes (Sutterwala *et al*., 2008).

In yeast, the sorting of glycosylphosphatidylinositol (GPI)-anchored proteins is influenced by sphingolipid- and ergosterol-rich lipid rafts (Zanolari *et al*., 2000), and thus yeast are dependent on *de novo* synthesis of ceramide (Sutterlin *et al*., 1997). This ceramide is synthesized in the ER and is transferred to the Golgi, by a process that is independent of ATP and vesicular trafficking (Funato and Reizman, 2001). The resulting IPC synthesized in the Golgi (Levine *et al*., 2000) is essential for stable membrane association of GPI-anchored proteins and subsequently their correct sorting and trafficking to the plasma membrane (Watanabe *et al*., 2002).

Sphingolipid/cholesterol-rich membrane lipid rafts of polarized mammalian epithelial cells have also been implicated to be associated with GPI anchors, which strongly influences the sorting of proteins leaving the Golgi (see reviews by Muniz and Reizman, 2000; Chatterjee and Mayor, 2001).

The abundant GPI-anchored variant surface glycoprotein (VSG) also strongly influences trafficking within the secretory and endocytic pathways in bloodstream-stage African trypanosomes (Engstler *et al*., 2004; Schwartz and Bangs, 2006). Inhibition of the initial step in the sphingolipid biosynthesis, even though essential for viability, surprisingly seems to have no detrimental effect on VSG trafficking to the cell surface (Sutterwala *et al*., 2007), despite reports of the presence of VSG in detergent-resistant membranes (Nolan *et al*., 2000; Denny *et al*., 2001). Thus, the extent to which lipid rafts might influence GPI-dependent trafficking in trypanosomes is still unclear (Denny and Smith, 2004).

Seminal work by the Bangs' group has resulted in the hypothesis that post-Golgi sorting of soluble and GPI-anchored cargo is influenced by the endocytic rate in each particular life cycle stage of *T. brucei* (Tazeh *et al*., 2009). This stage-specific variation is mirrored by the dependency of clathrin in endocytosis in the bloodstream stage (Allen *et al*., 2003), where the enlarged flagellar pocket, known as the ‘Big-Eye’ phenotype is observed as membrane vesicles continue to be delivered to the flagellar pocket, but none are internalized via the endocytic pathway from the flagellar pocket; the only site of endocytosis and exocytosis in these parasites (reviewed in Overath and Engstler, 2004).

Intracellular degradation of sphingolipids is achieved by SMases [EC. 3.1.4.12], which catalyses their hydrolysis to form ceramide and the corresponding headgroup i.e. phosphorylcholine in the case of SM.

Various SMases have been described, which differ in their subcellular localization, tissue specificity and are normally activated by growth factors, cytokines, chemotherapeutic agents, irradiation, nutrient removal and other related stresses (Tomiuk *et al*., 1998; Yabu *et al*., 2008). Some or all of these are thought to regulate the intracellular ceramide concentration and the corresponding stress-induced associated responses.

To date, there are five main types of SMases; the acidic Zn^2+^-dependent (cytosolic/secreted) and independent (lysosomal), the neutral Mg^2+^-dependent (membrane) and independent (cytosolic) and lastly the secreted alkaline SMase (reviewed in Goni and Alonso, 2002).

There is a growing body of evidence that suggests Mg^2+^-dependent neutral SMases (nSMases) are the major source for stress-induced ceramide production (Yabu *et al*., 2008). Ceramide and associated metabolites, such as sphingosine-1-phosphate, are known to function as second messengers, causing various biological activities in mammalian cells, including activation of protein-kinases and/or protein-phosphatases 2A (Huwiler *et al*., 2000). Increased levels of ceramides can exert anti-proliferation effects, induce apotosis and play major roles in mitogenesis and endocytosis.

Neutral SMases were first cloned from *Bacillus cereus* (Yamada *et al*., 1988), which allowed identification of nSMases in other bacteria including *Listeria ivanovii* (Gonzalez-Zorn *et al*., 1999). Through homology searches the yeast homologues (Sawai *et al*., 1999) and subsequently the mammalian homologues were identified (Tomiuk *et al*., 1998; Hofmann *et al*., 2000). Despite the extensive literature on nSMase activity it is only comparatively recently that mammalian nSMases have been cloned and characterized. Neutral SMases are ubiquitously expressed in mammalian cells, where they are predominately membrane-bound on the outer leaflet of the plasma membrane (Tomiuk *et al*., 1998), where most of the SM is located.

Plasmodium also contains a bacterial nSMase homologue, which is capable of cleaving both SM and lyso-PC (Hanada *et al*., 2002). Intriguingly, the compound scyphostatin inhibits this plasmodium enzyme activity, as well as preventing maturation from trophozoite to schizont at low µM concentrations (Hanada *et al*., 2000).

The yeast nSMase homologue Isc1 has been shown to be associated with the ER, during early growth, while in late logarithmic growth it is found in the outer leaflet of the mitochondria where it seems to regulate sphingolipid metabolism (Vaena de Avalos *et al*., 1998; Kitagaki *et al*., 2007). The Isc1 protein has inositol sphingolipid phospholipase C activity, i.e. capable of cleaving IPC to generate ceramide, which seems to be required for normal mitochondrial function (Sawai *et al*., 2000). Further recent studies have elaborated on this, by clearly showing that the knockout of Isc1 is essential during a diauxic shift, i.e. it plays a crucial role in the reprogramming of mitochondrial gene expression during the transition from anaerobic to aerobic metabolism, coupled with a change in carbon source (Kitagaki *et al*., 2009).

In this study, we begin to investigate the catabolism of sphingolipids in *T. brucei*.

Here we show the recombinant expression and characterization of the only *T. brucei* nSMase. We also report on the creation of a *T. brucei* conditional null mutant, which demonstrates that the nSMase activity is essential for the bloodstream form of the parasite, causing VSG trafficking to be impaired.

## Results

### Cloning and sequencing *T. brucei* nSMase

An nSMase homologue was putatively identified in the *T. b. brucei* (strain 927) genome database (Sanger Institute) by interrogating with the *Saccharomyces cerevisiae* nSMase (Isc1). This open reading frame (ORF) (Tb927.5.3710) was PCR-amplified from *T. b. brucei* strain 427, subcloned and sequenced. The sequence has been submitted to EMBL-EBI, Accession No. FM992873. An alignment of the predicted translated sequence with nSMases from other organisms is shown in Fig. S1A and an unrooted phylogenetic tree in Fig. S1B. The predicted 65 kDa *T. brucei* nSMase (*Tb*nSMase) has two putative transmembrane domains (Fig. S1A, underlined) towards the C-terminus, which are present in most eukaryotic nSMases, and contains the conserved residues critical for catalytic activity and Mg^2+^ binding (Fig. S1A, asterisks). Although *Tb*nSMase shows considerable similarity to nSMases from a variety of organisms, there are significant regions of the sequence, which are different between the kinetoplastid nSMase homologues (identified here) and the other nSMases including the human protein. Outside of the kinetoplasts the closest homologue is surprisingly the honey-bee (*Apis mellifera*).

### Overexpression of *Tb*nSMase in *E. coli*

**Fig. 1:**
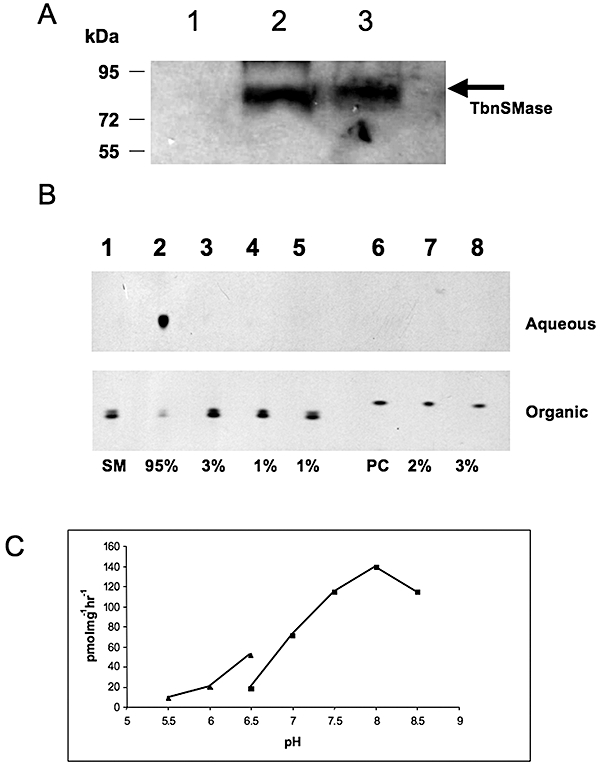
Expression, purification and activity of recombinant *Tb*nSMase. A. *Tb*nSMase was cloned and expressed as an N-terminal GST fusion protein in C43 *E. coli*. Protein samples after cell disruption were separated on a 10% SDS-PAGE gel, transferred to membrane and detected with anti-GST antibody. Lane 1, soluble protein (supernatant from 100 000 *g*); lane 2, membrane fraction (pellet from 100 000 *g*); lane 3, insoluble fraction (pellet from 45 000 *g*). B. The substrate [^3^H]-sphingomyelin (SM) and the product [^3^H]-choline–phosphate of the *Tb*nSMase assay were separated by phase partitioning (organic and aqueous respectively), analysed by HPTLC and detected by autoradiography as described in *Experimental procedures*. Lane 1, negative control, [^3^H]-SM with no membranes; lane 2, [^3^H]-SM with *Tb*nSMase; lane 3, [^3^H]-SM with *Tb*nSMase in presence of EDTA (5 mM); lane 4, negative control, [^3^H]-SM with non expressing *Tb*nSMase membranes; lane 5, [^3^H]-SM with non-expressing *Tb*nSMase membranes and EDTA (5 mM).; lane 6, [^3^H]-PC with no membranes; lane 7, [^3^H]-PC with *Tb*nSMase; lane 8, [^3^H]-PC with *Tb*nSMase in the presence of EDTA (5 mM). Percentages of substrate turnover are shown, as determined by densitometry (ImageJ software). C. *Tb*nSMase activity was determined by end-point radioactive assay as a function of pH, using with MES buffer (filled triangles) or Bis-Tris Propane (filled squares) as described in *Experimental procedures.*

To enable biochemical characterization, *Tb*nSMase was recombinantly expressed in *E. coli* using a pGEX-6P-1 vector. The N-terminal GST fusion protein (full-length, membrane bound) was expressed in *E. coli* C43 (DE3) cells, a bacterial host that allows the synthesis of potentially toxic membrane proteins (Miroux and Walker, 1996). The apparent molecular weight of this GST–*Tb*nSMase fusion protein was ∼81 kDa by Western blot analysis (Fig. 1A, lane 2), smaller than the predicted size of 91 kDa (including the GST tag). A significant proportion of the protein as expected was found in the membrane fraction (Fig. 1A, lane 3). These membranes were washed in phosphate-buffered saline (PBS) and suspended in 100 mM Tris (pH 7.4), 10 mM MgCl_2_ buffer containing 20% glycerol, and aliquots were stored at −80°C without loss of activity. Total protein content was calculated to be 50.8 µg µl^−1^ of this membrane preparation. Enzyme activity was assessed by incubation of GST–*Tb*nSMase containing membranes (127 µg protein) with either 25 nCi/50.5 pmol of [^3^H-choline-methyl] SM or 30 nCi/50.5 pmol of [^3^H-choline-methyl] PC, as mixed micelles with 0.1% Triton-X 100. Reactions were incubated at 37°C for 120 min in 100 mM Bis-Tris propane (pH 7.5), 5 mM MgCl_2_, 1% glycerol in the presence or absence of 5 mM EDTA. Chloroform/methanol/water phase separation was used to distinguish the radiolabelled lipid substrates from any aqueous radiolabelled products, which were subjected to scintillation counting and thin-layer chromatography. The assay showed that the membranes containing GST–*Tb*nSMase was catalytically active (Fig. 1B, compare lanes 1 and 2) and was also divalent cation-dependent (Fig. 1B, compare lanes 2 and 3). No activity towards PC was apparent (Fig. 1B, lanes 6–8), while no SMase activity was observed in equivalent *E. coli* membranes not containing GST–*Tb*nSMase (Fig. 1B, lanes 4 and 5). The optimum pH of the GST–*Tb*nSMase activity is pH 8, with very little activity at pH 5.5 (Fig. 1C), representative of an nSMase.

**Fig. 2:**
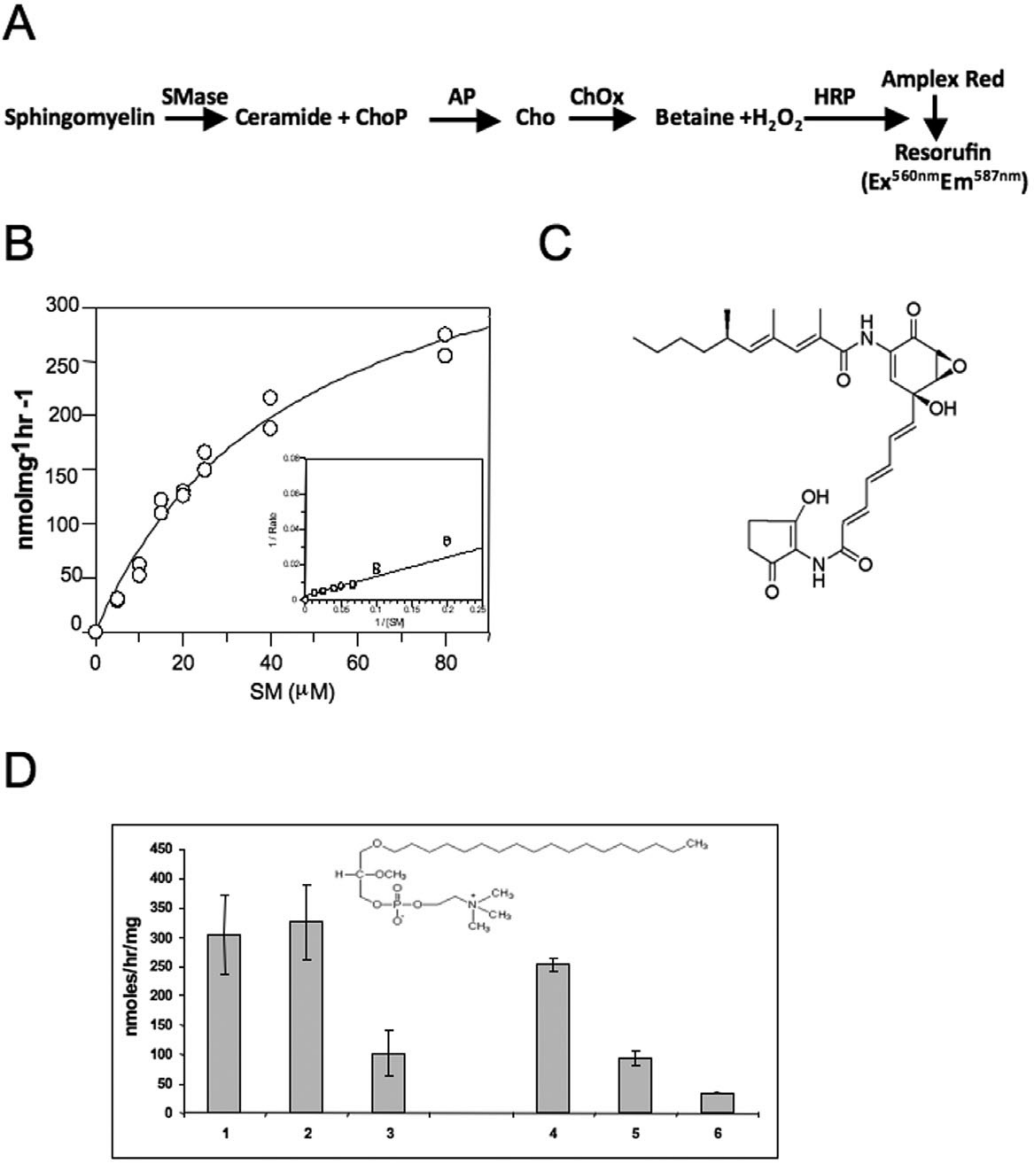
Activity of recombinant *Tb*nSMase. A. Reaction catalysed by *Tb*nSMase along with coupled the coupled Amplex red assay. AP, alkaline phosphatase; ChOx, choline oxidase; Cho-P, choline–phosphate; HRP, horseradish peroxidase. B. Determination of *Tb*nSMase Michaelis-Menten constants for SM (inserts show Lineweaver–Burk plot). C. Structure of manumycin A, a commercially available nSMase inhibitor. D. Enzyme activity of *Tb*nSMase in either washed bloodstream *T. brucei* membranes (lanes 1–3) or *E. coli* membranes expressing GST–*Tb*nSMase (lanes 4–6), after pre-incubation with either nothing (lanes 1 and 4) or miltefosine (lanes 2 and 5) or edelfosine (lanes 3 and 6) in the presence of SM as substrate as described in *Experimental procedures*. Insert shows structure of edelfosine.

Sphingomyelinase activity was similarly detected through the use of a multi-well plate, fluorescence-based, coupled assay (Invitrogen), whereby the product of SMase activity, choline–phosphate, is hydrolysed by alkaline phosphatase, and the released choline is oxidized to betaine and H_2_O_2_ (Fig. 2A). Production of H_2_O_2_ in the presence of horseradish peroxidase drives the conversion of the Amplex Red reagent to the red-fluorescent resorufin, detectable using a fluorescence microplate reader (Molecular Devices Gemini XPS). The *Tb*nSMase activity showed saturation kinetics were observed, with an apparent *K_m_* of 45.7 ± 6.5 µM and *V*_max_ of 424.0 ± 32.6 nmol h^−1^ mg^−1^ membrane protein (Fig. 2B).

Other homologous divalent cation-dependent nSMases assayed in a similar manner have similar kinetic properties including the following: (i) *S. cerevisiae* ISC1 (overexpressed in *S. cerevisiae* JK9-3dα cell lysates) with a *K_m_* of 34.8 µM and a *V*_max_ of 9200 nmol h^−1^ mg^−1^ (Sawai *et al*., 2000); and (ii) *Plasmodium falciparum* nSMase (GST fusion expressed in *E. coli* membranes) with an apparent *K_m_* of ∼90 µM and a specific activity of 5.69 ± 0.29 nmol h^−1^ mg^−1^ (Hanada *et al*., 2002). Three mammalian homologues have also been kinetically characterized: (i) rat nSMase (GST fusion partially purified from *E. coli*) with an apparent *K_m_* of 3.85 ± 0.73 µM and a *V*_max_ of 239.1 ± 33.2 nmol h^−1^ mg^−1^ (Mizutani *et al*., 2000); (ii) bovine nSMase (detergent extracted from bovine brain membranes) with an apparent *K_m_* of ∼40 µM and a specific activity of 371 nmol h^−1^ mg^−1^ (Bernardo *et al*., 2000); and (iii) human nSMase1 (His-nSMase expressed HEK293 cell lysates) with a *K_m_* of 26.2 ± 10.5 µM and a specific activity of 126.8 ± 17.4 nmol h^−1^ mg^−1^) (Sawai *et al*., 1999).

Sphingomyelinase activity, at a slightly reduced rate, was also detected using equivalent amounts of *E. coli* membranes expressing an N-terminal His-tagged version of the *Tb*nSMase protein (Fig. S2), while again no activity was detected towards PC (Fig. S2, lane 3). The fluorescence-based coupled assay is amenable to high-throughput screening for inhibitors of the recombinant *Tb*nSMase. This allowed the determination of IC_50_ values of various compounds including known nSMase inhibitors such as the dihydroimidazolo-amide GW4869 (Luberto *et al*., 2002) and Manumycin A (Fig. 2C) (Arenz *et al*., 2001), which gave IC_50_ values of 14.4 ± 2.3 µM and 24.6 ± 4.6 µM respectively. Manumycin A also has an ED_50_ of 10.0 ± 6.5 µM; however, it should be noted that it has previously been shown to be trypanocidal, with the suggestion of it being a farnesyl transferase inhibitor (Ali *et al*., 1999), in light of our findings that it may be that Manumycin A has multiple trypanocidal targets. The *lyso*-PC analogues miltefosine and edelfosine (structure insert Fig. 2D) are also inhibitors of the nSMase activity in *T. brucei* membranes (Fig. 2D, lanes 1–3) and recombinant GST–*Tb*nSMase activity (Fig. 2D, lanes 4–6). Edelfosine is more potent that miltefosine, such that it has an IC_50_ of 21.0 ± 3.2 µM against the recombinant GST–*Tb*nSMase (Fig. 2D, lane 6).

### nSMase is located in the endoplasmic reticulum in the bloodstream form of *T. brucei*

**Fig. 3:**
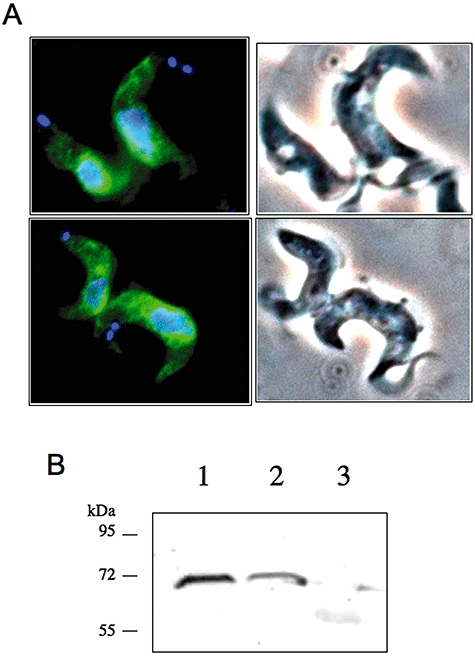
Localization of *Tb*nSMase*-HA*^Ti^ in bloodstream form *T. brucei*. A. Fixed *Tb*nSMase*-HA*^Ti^ cells were incubated with rat anti-HA antibody, and rabbit anti-rat FITC conjugated antibody and DNA stained with DAPI. B. Anti-HA Western analysis of fractions from differential centrifugation, 14 500 *g* pellet (1), 140 000 *g* pellet (2) and supernatant of 140 000 *g* pellet (3) of *Tb*nSMase*-HA*^Ti^ cells, prepared as described in the *Experimental procedures*.

The cellular location of *Tb*nSMase was investigated using the tetracycline-inducible overexpression vector pLew82, containing a full-length *Tb*nSMase with a C-terminal HA-tag. This construct was introduced into bloodstream form *T. brucei* cells, generating the cell line *Tb*nSMase*-HA*^Ti^ and integration was confirmed by PCR using specific vector primers (data not shown). Tetracycline-induced *Tb*nSMase-*HA*^Ti^ cells were fixed, adhered to poly-lysine slides and permeablized. The *Tb*nSMase-*HA*^Ti^ protein was detected by immunofluorescence as a perinuclear staining with some reticular cytoplasmic signal (Fig. 3A and Fig. S3), suggesting that *Tb*nSMase is localized in the ER. This was confirmed by subcellular fractionation studies, where the membrane bound *Tb*nSMase*-HA* protein was detected in the microsomal fraction and not the cytosolic fraction (Fig. 3B).

### *Tb*nSMase is an essential gene in the bloodstream form of *T. brucei*

**Fig. 4:**
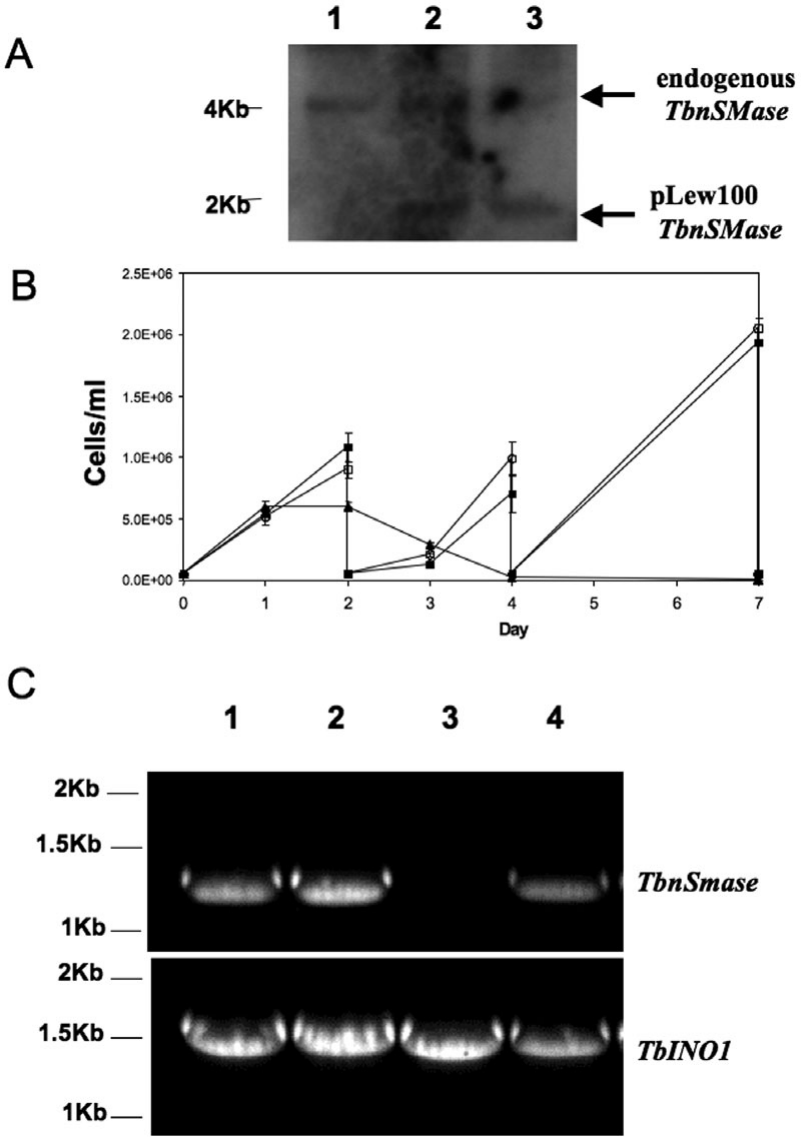
*Tb*nSMase is essential for the survival of bloodstream form *Trypanosoma brucei* in culture. A. Confirmation of genotype of *T. brucei Tb*nSMase conditional double knockout cell line. Southern blot analysis of *Pst1*-digested genomic DNA (3 µg); the *Tb*nSMase ORF probe shows allelic *Tb*nSmase at 4 kb and the ectopic copy *– Tb*nSMase*-HA^Ti^* at ∼2 kb; parental cells (lane 1); ΔnSMase:*::PAC/Tb*nSMase*-HA^Ti^* (lane 2); ΔnSMase:*::PAC*ΔnSMase:*::HYG/Tb*nSMase*-HA^Ti^* (lane 3). B. Growth curves of *T. brucei* parental cells (1 – filled squares) and *Tb*nSmase conditional knockout cells grown in the presence (2 – empty circles) or absence (3 – filled triangles) of tetracycline. C. RT-PCR amplification of *Tb*nSMase RNA transcripts from total RNA extracted from wild-type cells (lane 1) and *Tb*nSMase conditional null mutants either grown in the presence (lane 2) or absence of tetracycline for 1 and 10 days (lanes 3 and 4 respectively). The upper panel shows RT-PCR products using primers specific for *Tb*nSMase; the lower panel shows a loading control using (*TbINO1*) primers.

Southern blot analysis of *T. brucei* genomic DNA showed that *Tb*nSMase is present as a single copy gene per haploid genome (data not shown), and RT-PCR analysis clearly shows that the *Tb*nSMase is expressed in both bloodstream and procyclic forms of the parasite (Fig. S4), thus making it amenable to genetic validation as a drug target. One allele of *Tb*nSMase was replaced with a knockout cassette containing the puromycin drug resistance gene, by homologous recombination and selection with puromycin, creating the Δ*Tb*nSMase*::PAC* cell line (Fig. 4A, Lane 1). Attempts to create a null mutant by homologous replacement of the second allele with the hygromycin resistance gene were unsuccessful. Therefore, as the ‘wild-type’ cell line used here constitutively expresses the T7 RNA polymerase and the tetracycline repressor protein, a tetracycline-inducible (Ti) *HA-*tagged ectopic copy of the *Tb*nSMase was integrated in the rDNA locus (Wirtz *et al*., 1999) via a modified version of the pLew100 vector encoding the blasticidin drug resistance protein (previously constructed in the Smith lab) forming the *Tb*nSMase-*HA*^Ti^Δ*Tb*nSMase*::PAC* cell line (Fig. 4A, Lane 2). The second allele was replaced with the hygromycin resistance gene in the presence of tetracycline. A *Tb*nSMase-*HA*^Ti^Δ*Tb*nSMase*::PAC/*Δ*Tb*nSMase*::HYG* clone was obtained and the genotype confirmed by Southern blot analysis (Fig. 4A, Lane 3). To establish the essentiality of *Tb*nSMase in bloodstream *T. brucei*, the growth of the *Tb*nSMase-*HA*^Ti^Δ*Tb*nSMase*::PAC/*Δ*Tb*nSMase*::HYG* conditional knockout (cKO) cell line was monitored in the absence and presence of tetracycline in certified tetracycline-free HMI-9. In the presence of tetracycline the cKO cell line showed normal growth rates when compared with wild-type cells (Fig. 4B). However, in the absence of tetracycline, the cells grew normally for ∼24–28 h before ceasing division and steadily declined in cell numbers to below the limits of detection by light microscopy by day 5. At day 10 however, some live cells were visualized having wild-type morphologies that subsequently resumed normal growth kinetics. RT-PCR analysis showed that the transcript level of the ectopic *Tb*nSMase*-HA*^Ti^ was similar to that of the endogenous copy seen for wild-type cells (Fig. 4C, compare lanes1 and 2). In the absence of tetracycline for 24 h the transcript of *Tb*nSMase*-HA*^Ti^ was undetectable (Fig. 4C, lane 3), whereas in the revertant cells, a transcript for the *Tb*nSMase*-HA*^Ti^ is clearly shown (Fig. 4C, lane 4), suggesting that these revertant cells overcome the tetracycline control, a phenomena that has been described for other essential genes in *T. brucei* (Chang *et al*., 2002; Roper *et al*., 2002; Martin and Smith, 2006a).

### Lipidomic analysis of the nSMase conditional knockout

The biochemical phenotype of the *Tb*nSMase conditional knockout cells was first investigated using mass spectrometry to examine differences in phospholipid species between the wild-type and nSMase conditional knockout trypanosomes. Based on the growth curve (Fig. 4B), 42 h without tetracycline was chosen as the optimum time point to characterize the *Tb*nSMase cKO phenotype, as the cells were still viable and metabolically active. Six hours later (48h–tet) this is not the case, thus 42 h was deemed the most extreme point at which to investigate the advanced phenotype without studying dying cells.

Initially, ES-MS was used to examine the phospholipid content of whole-cell extracts from *Tb*nSMase-depleted cells, focussing initially on the positive ion mode for detection of both PC and SM. However, no differences in the parent species of the collision-induced daughter ion characteristic of PC and SM phospholipids (parents of 184 m/z) were observed between wild-type and cKO cells cultured in the absence of tetracycline for 42 h (*Tb*nSMase cKO (42h–tet)) (Fig. S5). Also only very minor changes were detected in the corresponding negative ion survey scans (Fig. S6A). Parent ion and neutral loss scanning of individual phospholipid classes were acquired for PI, phosphatidylethanolamine (PE), phosphatidylserine (PS) (Fig. S6B–D). The only difference of any note when compared with wild-type bloodstream form phospholipid spectra was the unexpected presence of inositolphosphorylceramide (IPC) (34:1, 778m/z) (Fig. S6B, arrow).

**Fig. 5:**
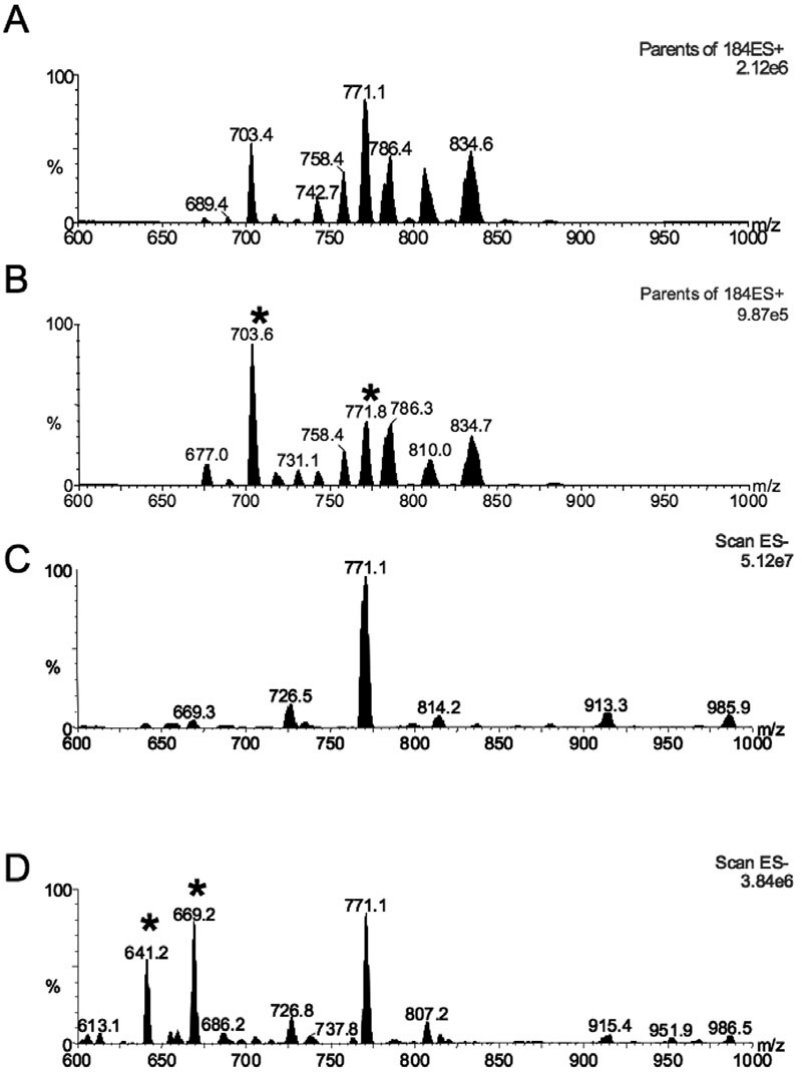
Mass spectrometric analyses of phospholipids. Choline-containing phospholipids extracted from the P100 fraction as described in *Experimental procedures* were analysed by ESI-MS/MS in positive ion mode using parent-ion scanning of the collision induced fragment for phosphorylcholine at 184 m/z in (A) parental cells. (B) *Tb*nSMase cKO cells grown in the absence of tetracycline for 42 h. ESI-MS negative ion survey scan spectra of P100 lipid extracts from *T. brucei* bloodstream form cells. (C) Parental cells. (D) *Tb*nSMase cKO cells grown in the absence of tetracycline for 42 h. Asterisks highlight ions, which are altered significantly in intensity, as described in the text

Consequently, it was hypothesized that any potential differences in the phospholipid profiles may be in a particular organelle such as the ER. Thus, lipids were extracted from a microsomal fraction (100,000g pellet, P100), representing internal organelle membranes, from both wild-type and *Tb*nSMase cKO (42h–tet) trypanosomes and analysed by ES-MS and ES-MS-MS. Upon collision-induced daughter ion scanning for choline–phosphate phospholipids (parents of 184 m/z), i.e. PC and SM (Fig. 5A and B), differences were observed. These included a significant decrease in the PC species at 771 m/z (alkylacyl 36:2), as well as minor decreases in PC species at 807 m/z (diacyl C38:5) and 835 m/z (diacyl C40:5) (Fig. 5A and B, species highlighted with asterisks). This decrease is in contrast with the significant increase in the SM species at 703 m/z (C16:0) and minor increases in the SM species at 677 m/z (C14:0) and 731 m/z (C18:0). Fully characterization of the choline–phosphate containing phospholipids in *T. brucei* is shown in Table S1 (Richmond *et al*., 2010).

In contrast, the negative ion survey scan of the P100 subcellular membrane fraction of wild-type cells (Fig. 5C) only showed one major species at 771 m/z, identified by ES-MS/MS daughter ion fragmentation (Fig. S7A) as phosphatidylglycerol (PG) (diacyl, C36:3, a mixture of C18:1, C18:2 and C18:0, C18:3). The corresponding negative ion survey scan of the *Tb*nSMase cKO (42h–tet) P100 fraction (Fig. 5D), also showed the PG (diacyl, C36:3) species to be the major species; however, there was a significant increase in two species at 641 m/z and 669 m/z (Fig. 5D, marked with an asterick), identified by ES-MS/MS daughter ion fragmentation (Fig. S7B), as diacylglycerol (DAG) species C38:5 (C16:0, C22:5) and C40:5 (C18:0, C22:5) respectively. As with the whole-cell extracts, the presence of IPC (34:1, 778m/z) was observed in the *Tb*nSMase cKO (42h–tet) P100 fraction of inositol-phosphate containing phospholipids (parents of 241 m/z) (Fig. S7C). IPC is normally not present in bloodstream form *T. brucei*, but is in procyclic and stumpy form parasites (Güler *et al*., 2008; Kabani *et al*., 2009).

Taken together, this set of data clearly shows an increase of SM species at the organelle level due to the lack of nSMase activity. The corresponding decrease in PC is explainable by a lack of the choline–phosphate product from SM hydrolysis by *Tb*nSMase in the ER, thus reducing the flux through the Kennedy pathway of *de novo* PC biosynthesis. Alternatively, or most likely concurrently, SM is being synthesized at the expense of depleting the pool of PC by the SM synthases.

### Loss of nSMase activity leads to a ‘Big-Eye’-like phenotype

**Fig. 6:**
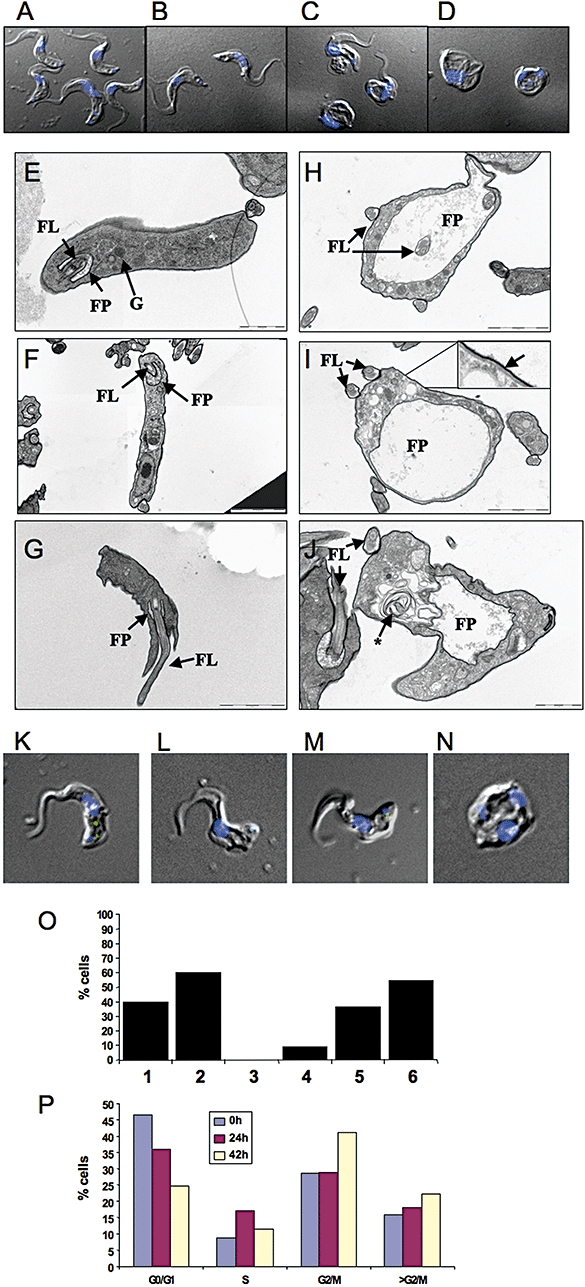
Morphological phenotype of the *Tb*nSMase conditional knockout. A–D. Merged DAPI-fluorescence-DIC images of wild-type (A), and *Tb*nSMase cKO cells grown in the absence of tetracycline for 42 h (B–D). E–J. Transmission electron micrographs taken of wild-type bloodstream *T. brucei* (E–G) and *Tb*nSMase cKO cells grown in the absence of tetracycline for 42 h (H–J). Insert in (I) shows a close up of the plasma membrane, microtubles and the fussy VSG coat. FL, flagellum; FP, flagellar pocket; K, kinetoplast; M, mitochondria; G, glycosome; The asterisk in (J) indicates that enlarged concentric membrane structure may be part of the endosomal network. K–O. Wild-type (K) and *Tb*nSMase cKO cells grown in the absence of tetracycline for 42 h (L–N) were incubated with FITC-BSA as described in *Experimental procedures*; images are merged FITC, DAPI-fluorescence and DIC. (O) Evaluation of the endocytic function of wild-type (1–3) and nSMase cKO (Tet-42h) (4–6) trypanosomes were assessed for the incidence of the FITC-BSA signal either solely in the endosomal region (1 and 4), or dual endosomal and flagellar pocket staining (2 and 5) or flagellar pocket staining only (3 and 6). P. FACS analysis of *Tb*nSMase cKO cells grown in the absence of tetracycline for 0, 24 and 42 h. Cells were fixed, stained with propidium iodide and analysed by flow cytometry as described in *Experimental procedures*.

The *Tb*nSMase cKO cell line grown in the absence of tetracycline exhibited a phase light structure at the posterior end, which significantly increases in volume with time. This enlarged structure eventually becomes the major proportion of the cell volume and the trypanosomes become rounded with time (Fig. 6B–D) compared with wild-type cells (Fig. 6A). Concurrently, the proportion of these abnormal cells increased through time such that at 42 h, ∼25% of nSMase cKO cells exhibit this morphology, increasing to over 50% of the cells at 72 h, compared with <1% of wild-type cells. This morphology is similar to the ‘Big-Eye’ phenotype described previously with the RNA interference (RNAi) of the clathrin heavy chain in *T. brucei* bloodstream forms (Allen *et al*., 2003). Allen and colleagues used immunogold α-VSG electron microscopy to confirm that the phase light structure was the flagellar pocket, massively enlarged from the small flask-shaped structure usually seen in wild-type cells.

Electron microscopy of the *Tb*nSMase cKO grown under permissive conditions also showed the highly enlarged flagellar pocket (Fig. 6H–J), when compared with the wild-type cells (Fig. 6E–F, further EM micrographs in Fig. S8).

This ‘Big-Eye’ enlargement of the flagellar pocket can be attributed to a defect in membrane transport whereby the balance of exocytosis and endocytosis are disturbed.

The decrease in fluid endocytosis in *Tb*nSMase cKO (42h–tet) cells (Fig. 6L–N), compared with wild-type cells (Fig. 6K), was confirmed with FITC-BSA uptake by immunofluorescence. The FITC-BSA signal can be observed frequently in the endosomal network and occasionally at the flagellar pocket in wild-type cells (Fig. 6K), but is significantly more frequently associated with just the flagellar pocket in the *Tb*nSMase cKO (42h–tet) cells; only a fraction is endocytosed after 30 min (Fig. 6O, lanes 4–6), compared with a significant portion of the FITC-BSA being endocytosed in the wild-type cells (Fig. 6O, lanes1–3) (Fisher's exact test, *P* = 0.056, *n* = 40 fields). Extreme morphological ‘Big-Eye’ trypanosomes typically demonstrated no specific FITC staining even with longer camera exposures (Fig. 6H).

As with the clathrin RNAi ‘Big-Eye’ trypanosomes, *Tb*nSMase cKO (42h–tet) cells could be seen to be undergoing mitosis and sometimes presented with two phase-light structures (Fig. 6C, D and N), one larger flagellar pocket confirmed to be the original, and a smaller daughter flagellar pocket exocytically active prior to cytokinesis (Allen *et al*., 2003). Associated with this ‘Big-Eye’ phenotype was a noticeable increase in pre-cytokinesis 2K2N cells. This lack of proper cell-division of the *Tb*nSMase cKO (-tet) was confirmed by FACS analysis, which showed a significant increase in the proportion of G2/M, with a corresponding decrease in G0/G1 cells (Fig. 6P), implying a cell cycle arrest at G2/M.

To ensure that the enlargement of the flagellar pocket in this cell line is similar to the previously described ‘Big-Eye’ defect in endocytosis phenotype, several previously well-characterized proteins that are sorted by the endosomal network were investigated.

### Does the loss of nSMase affect protein trafficking?

**Fig. 8:**
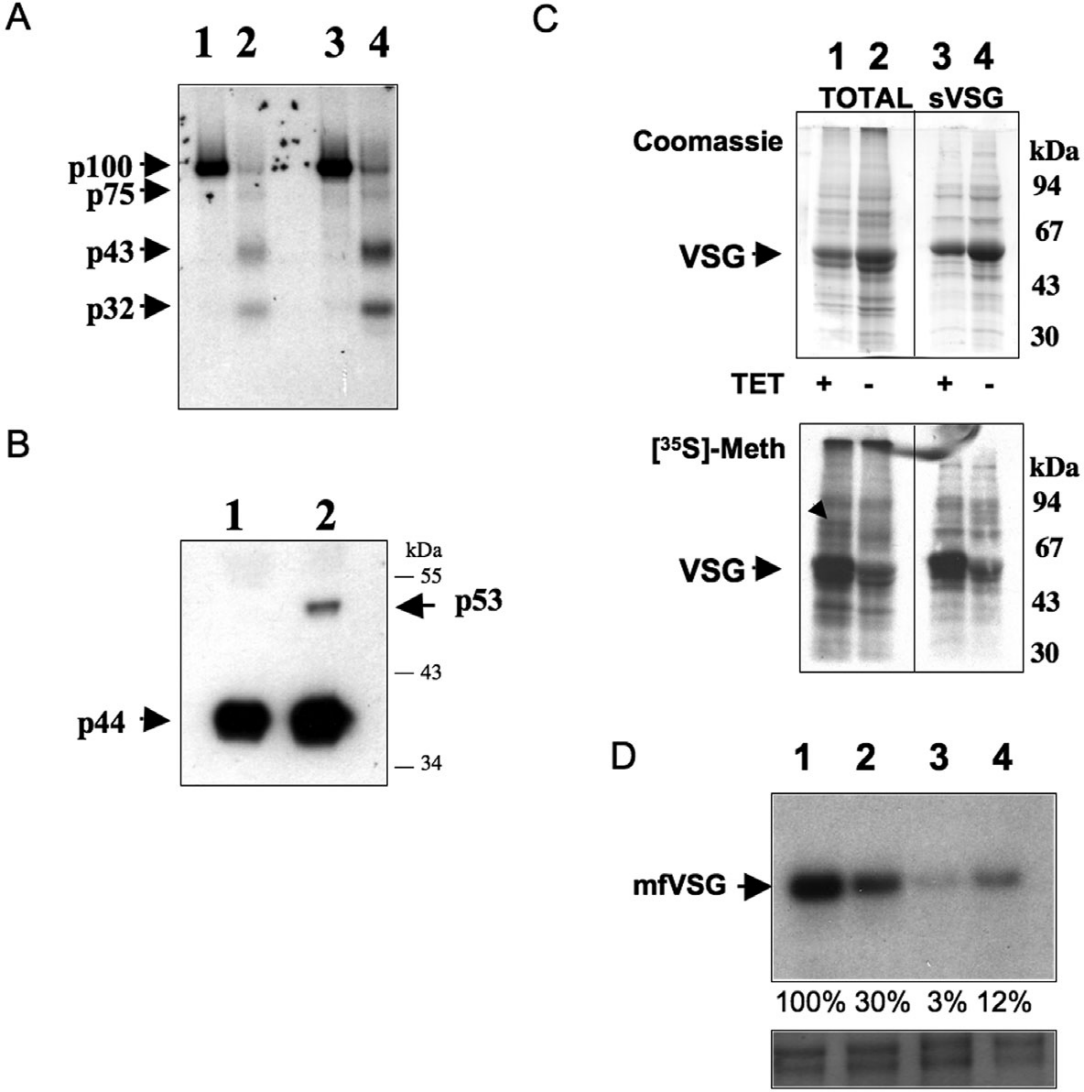
Phenotypic analysis of protein trafficking in the nSMase conditional knockout. A. Wild-type and *Tb*nSMase cKO cells grown in the absence of tetracycline for 42 h were pulse labelled with [^35^S]-methionine for 1 h (lanes 1 and 3 respectively) and chased for further 2 h (lanes 2 and 4 respectively) prior to IP with anti-P67 as described in *Experimental procedures*. The resulting IP [^35^S]-proteins was separated on a 10% SDS-PAGE gel, prior to Coomassie blue staining (Fig. S6) and fluorography. The various maturation sizes of P67 are highlighted. B. Anti-trypanopain Western blot analysis of wild-type (lane 1) and *Tb*nSMase cKO cells grown in the absence of tetracycline for 42 h (lane 2) shows incomplete endosomal processing. The maturation sizes of trypanopain are highlighted. C. Wild-type (lanes 1 and 3) and *Tb*nSMase cKO cells grown in the absence of tetracycline for 42 h (lanes 2 and 4) were labelled with [^35^S]methionine for 1 h. Total [^35^S]-protein (lanes 1 and 2) or [^35^S]-sVSG (soluble VSG-obtained from the cell surface of the parasite) (lanes 3 and 4) prepared as described in *Experimental procedures* were separated on a 10% SDS-PAGE gel, and visualized either by Coomassie blue or fluorography. The black arrow (lane 1) refers to a protein that is no longer being synthesized in the *Tb*nSMase cKO (-tet) cells (lane 2). D. Wild-type (lanes 1 and 2) and *Tb*nSMase cKO cells grown in the absence of tetracycline for 42 h (lanes 3 and 4) were labelled with [^3^H]myristate for 1 h in the absence (lanes 1 and 4) or presence of cycloheximide (60 µg ml^−1^) (lanes 2 and 3) as described in *Experimental procedures.* Densitometry (ImageJ) of the [^3^H]myristate-mfVSG (membrane form VSG, processes an intact GPI-anchor) signals is normalized to 100% for the wild-type cells in the absence of cycloheximide. Lower panel shows the VSG portion of the gel stained with Coomassie blue as loading control.

As a diagnostic marker for membrane protein trafficking in bloodstream form *T. brucei*, the processing of p67 in *Tb*nSMase cKO cells was examined. It is well documented that the terminal endosomal marker p67, a type I membrane protein, undergoes complex processing in bloodstream form trypanosomes (Brickman and Balber, 1994). From its synthesis as an ER-associated 100 kDa glycoprotein (gp100), p67 is further N-glycosylated to a 150 kDa species (gp150) in the Golgi, before being transported and proteolytically cleavage in the lysosome, forming firstly gp75 and subsequently gp28, gp32, gp42. Using the method of Alexander *et al*. (2002), this processing can be monitored. Thus, wild-type and *Tb*nSMase cKO (42h–tet) cells pulse labelled for 1 h with [^35^S]-methionine were immunoprecipitation at 0 and 2 h post pulse, using the monoclonal α-p67 (mAb280 – a gift of J. Bangs). Analysis of the [^35^S]-methionine-labelled immunoprecipitates by SDS-PAGE and autoradiography revealed that proteolytic processing in the p67 through the gp75 intermediate to the end products gp32 and gp42 was unaffected by the lack of *Tb*nSMase (Fig. 8A, compare lanes 1 and 2, with 3 and 4, loading controls Fig. S9).

Similarly, the trafficking of lumenal proteins in *Tb*nSMase cKO cells was investigated by examining the processing of trypanopain, a cathepsin L orthologue. In wild-type bloodstream form trypanosomes, trypanopain is synthesized as a 53 kDa pro-protein and rapidly fully processed in the acidic environment of the endosomal network into a 44 kDa mature active enzyme (Caffrey *et al*., 2001), similarly observed by ourselves using Western blot analysis with α-trypanopain Ab (a gift of J. Bangs) (Fig. 8B, lane 1). However, in *Tb*nSMase cKO (42h–tet) cells, proteolytic processing of trypanopain is incomplete with 2–5% of the immature 53 kDa pro-enzyme detectable by Western blot (Fig. 8B, lane 2).

**Fig. 7:**
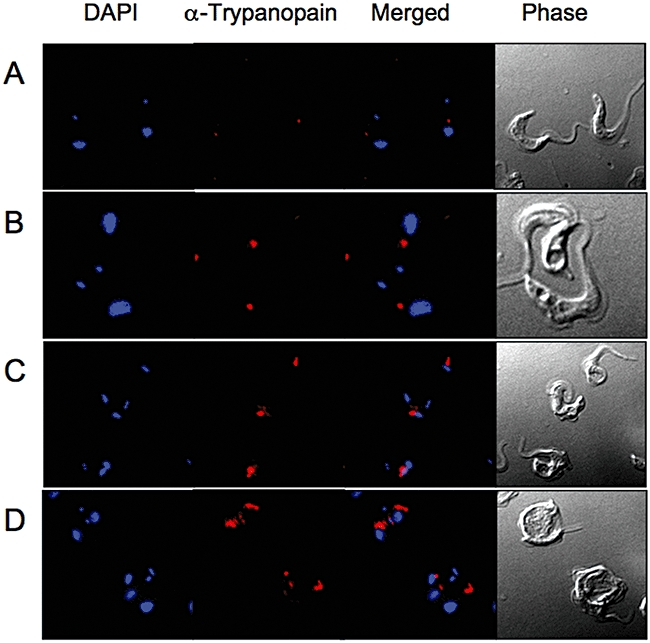
Investigation of the endosomal network. Wild-type cells (A) and *Tb*nSMase cKO cells grown in the absence of tetracycline for 42 h (B–D) probed with anti-trypanopain. Images are of TRITC, DAPI-fluorescence and DIC.

Examination in *Tb*nSMase cKO cells (42h–tet) of the subcellular localization of trypanopain, which typically colocalizes with p67 in the lysosome (Caffrey *et al.*, 2001), shows that the endosomal staining pattern is greatly enlarged (Fig. 7B–D and Fig. S10) compared with the corresponding wild-type staining pattern (Fig. 7A). In addition, extension of the endosomal staining is congruent with the expansion of the late endosomal network and flagellar pocket. This indicates that the endosomal network is malfunctioning, probably as a result of lack of flux through the endocytic pathways.

This malfunctioning of the endosomal network may account for the unusual and, as far as we know, novel structures observed in a few of the electron microscopy sections of the *Tb*nSMase cKO cells (42h–tet) (Fig. 6I and J and Fig. S8F and G) consisting of concentric ‘onion-like’ vesicular membranes.

Another soluble protein that is found in the endocytic pathway, protein disulphide isomerise 2 (PDI-2) (Rubotham *et al*.*,* 2006), was similarly examined for any aberrant processing. As a 55 kDa polypeptide, PDI-2 is subsequently heavily glycosylated into an 80 kDa glycoprotein in the Golgi, and ultimately colocalizes with p67 in the terminal endosomes. Western blot analysis of wild-type and *Tb*nSMase cKO (42h–tet) cells with α-PDI-2 (a gift from Derek Nolan) revealed equal amounts of the ∼80 kDa glycoprotein species (Fig. S11), indicating no drastic defect in processing.

### Does the loss of nSMase affect VSG trafficking?

To investigate the effect, if any, on exocytosis of newly synthesized VSG reaching the flagellar pocket, cells were labelled with [^35^S]-methionine for 1 h, after which total protein and soluble VSG (sVSG) preparations (obtained by GPI-PLC cleavage of cell surface VSG only upon mild hypotonic lysis) were separated by SDS-PAGE and [^35^S]-labelled proteins detected by autoradiography. Note: equal cell equivalents were loaded on the gel, but due to the ‘Big-Eye’ 2K2N pre-cytokinesis phenotype, the actual amount of total protein is 1.4 times higher in the *Tb*nSMase cKO (42h–tet) as determined by Bradford and shown in Fig. 8C (lanes 1 and 2). The total amount of [^35^S]-methionine incorporation into protein (excluding VSG) between the cell lines is similar (Fig. 8C, lanes 1 and 2). Thus at this 42 h time point, *Tb*nSMase cKO cells were essentially viable based on their ability to synthesize protein at a level comparable to wild-type cells.

However, it is clear that the proportion of newly synthesized ([^35^S]-methionine labelled) VSG exported to the cell surface, i.e. the radiolabelled sVSG, is drastically reduced in the *Tb*nSMase cKO (42h–tet) cells, when compared with the Coomassie stained sVSG (Fig. 8C, lanes 3 and 4) and the α-VSG221 Western blot analysis (Fig. S9B), thus one can conclude that the rate of newly synthesized VSG getting to the cell surface has been drastically reduced in the *Tb*nSMase cKO (42h–tet) cells. The protein bands other than sVSG have very similar amounts of [^35^S]-methionine incorporation, showing that protein synthesis was still taking place. The exception to this is a ∼80 kDa protein (Fig. 8C, lane 1, black arrow), the corresponding protein band that was identified by mass fingerprinting as possibly either HSP-70 or PDI-2, interestingly, both of which are involved in protein folding.

To investigate this defect in VSG synthesis, and its subsequent exocytosis, both wild-type and *Tb*nSMase cKO (42h–tet) cell lines were labelled with [^3^H]-myristate. As expected in wild-type cells the mature membrane form GPI-anchored VSG (mfVSG) was [^3^H]-myristate labelled (Fig. 8D, lane 1). The corresponding [^3^H]-myristate labelled mfVSG in the *Tb*nSMase cKO (42h–tet) cells however was significantly reduced (∼12%) (Fig. 8D, lane 4). This significant reduction prompted an assessment of the myristate exchange of the GPI anchor of pre-existing VSG, which takes place during endocytosis. As previously shown, in the presence of cycloheximide (to inhibit protein synthesis), the myristate exchange in wild-type cells shows a residual ∼30% (Fig. 8D, lane 2) [^3^H]-myristate incorporation (Buxbaum *et al*., 1996). However, in the *Tb*nSMase cKO (42h–tet) cells the incorporation is reduced to a mere ∼3% compared with wild-type level. Despite this significantly reduced amount of myristate exchanging happening in *Tb*nSMase cKO (42h–tet) cells, it is approximately the same ratio of myristate exchange to total myristylated mfVSG observed in wild-type cells, i.e. 3–12% and 30–100% respectively. This indicates that despite the rate of endocytoses and exocytoses of mfVSG being significantly reduced their ratio (relationship) is maintained.

## Discussion

The essentiality of the *Tb*nSMase clearly shows that the *de novo* synthesis of ceramide cannot compensate for the loss of ceramide formation via SM catabolism in the ER.

The nSMase cKO trypanosomes grown under permissive conditions also shows a significant increase in intracelluar SM levels, either in the ER and/or the mitochondria where a SM synthase is present. In conjunction with the excess SM is a significant decrease in PC, and a corresponding increase in intracelluar DAG. The observed increase in DAG species of the same diacyl chain length as the depleted PC species (C38:5 and C40:5) implies that the *de novo* synthesis of PC via the Kennedy pathway has slowed, whereas the synthesis of DAG has not, resulting in excess DAG. Alternatively, or more likely concurrently, the rate of SM synthesis continues unabated resulting in increased SM levels and the by-product DAG, which is not being utilized or catabolized.

The depletion of the PC in the mitochondrion comes about because the cells become starved of choline and/or choline–phosphate as the local pool of choline is tied up as SM. The absence of the nSMase in the ER means that no choline–phosphate is liberated, which could be utilized to synthesize PC via the Kennedy pathway. As the parasite is auxotrophic for choline, it must source choline containing phospholipids from the host environment, probably primarily as components of low-density-lipoprotein (LDL). However, when the nSMase cKO is grown under permissive conditions, it has a greatly reduced endocytic flux, ultimately preventing the catabolism of both pre-exisiting endosomal SM or PC, and if any, freshly endocytosed extracellular SM or PC.

A consequence of this shift in choline usage is that the mitochondrial associated SM synthase ultimately runs out of its normal substrate, PC, and starts to utilize PI instead, thus giving rise to this unusual IPC formation in bloodstream form *T. brucei*. This pool of PI in the ER is derived from the *de novo* synthesized inositol (Martin and Smith, 2006b). This implies that a mitochondrial SM synthase can utilize both PC and PI as substrates for the synthesis of SM and IPC respectively. However, in the bloodstream form of the parasite it prefers to utilize PC, and only uses PI if no PC is available. This is in contrast to procyclic cells where substantial amounts of IPC have been observed in purified mitochondria, despite there being ample PC available (Güler *et al*., 2008).

An alternative possibility for the observed IPC formation is that the *de novo* synthesized ceramide is translocated to the Golgi where the resident SM synthase (SLS4), known to be capable of utilizing both PC and PI (Mina *et al*., 2009), now starved of PC utilizes a different pool of PI, which is synthesized in the Golgi from the extracellular inositol (Martin and Smith, 2006a). The transition of PC to PI utilization as substrates by mitochondrial SM synthase(s) also occurs during bloodstream long-slender to stumpy form differentiation, where IPC formation is clearly observed along with dramatic upregulation of a mitochondrial associated SM synthase (SLS1) (Kabani *et al*., 2009). These observations imply that both stumpy and procyclic form parasites have a significantly decreased rate of ceramide formation in the ER, as there is little, if any, SM for the nSMase to catabolize, and thus the flux of post-Golgi sorting of protein destined for the recycling endosomes and flagellar pocket, which is dependent upon ceramide from the ER, would be severely reduced.

**Fig. 9:**
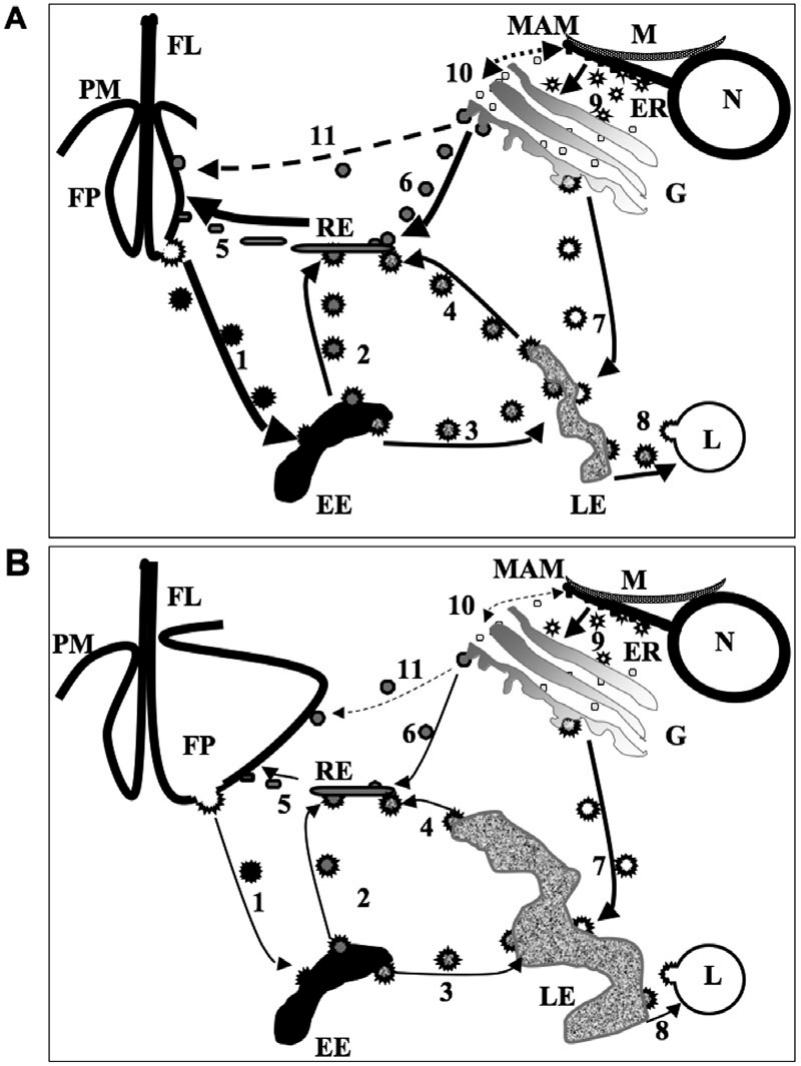
Schematic of the pathways involved in endocytosis and exocytosis in bloodstream form *T. brucei*: wild-type (A) and the nSMase cKO grown under permissive conditions (B). The difference in the width of the arrows in (A) and (B) depicts the relative changes in the flux for the same vesicular trafficking pathway in the wild-type cells compared with the nSMase cKO grown under permissive conditions. Dashed arrows indicate putative pathways. The enlarged flagellar pocket and late endosomes are representative of the ‘Big-Eye’ phenotype observed in the nSMase cKO grown under permissive conditions (B). Annotated cellular structures: EE, early endosomes; ER, endoplasmic reticulum; FP, flagellar pocket; G, Golgi; L, lysosome; LE, late endosomes; M, mitochondria; MAM, mitochondrion associated membrane; PM, plasma membrane; RE, recycling endosomes; FL, flagellum. The various types of vesicles are numbered as follows: 1. Endocytic clathrin-coated vesicles from flagellar pocket to early endosomes, carrying a mixture of endocytosed nutrients, including LDL particles and transferrin bound to GPI-anchored transferrin receptor, and recycling pre-existing cell surface GPI-anchored VSG, to be checked and undergoing myristate exchange. 2. Clathrin-coated vesicles from early endosomes to recycling endosomes carrying recycling pre-existing cell surface GPI-anchored VSG. 3. Clathrin-coated vesicles from early endosomes to late endosomes, carrying a mixture of endocytosed nutrients, including LDL particles and transferrin bound to GPI-anchored transferrin receptor. 4. Clathrin-coated vesicles from late endosomes to recycling endosomes, carrying the recycled GPI-anchored transferrin receptor, now free of transferrin, and newly synthesized membrane-bound protein destined for the flagellar pocket, not newly synthesized GPI-anchored proteins, i.e. VSG. 5. Non-clathrin-coated, sphingomylein-enriched recycling endosomes destined for the flagellar pocket, which contains recycling GPI-anchored transferrin receptor, recycling VSG and newly synthesized VSG. 6. Non-clathrin-coated, SM-enriched vesicles from the *trans*-Golgi cisterna to the recycling endosomes, containing newly synthesized VSG and possibly other GPI-anchored proteins, ultimately destined for the flagellar pocket and the cell surface. 7. Clathrin-coated vesicles from the Golgi to late endosomes, containing newly synthesized proteins which will be targeted to go either to the lysosome, i.e. p67 and trypanopain, or to go via the recycling endosomes to the flagellar pocket, i.e. cell surface proteins other than VSG. 8. Clathrin-coated vesicles from the late endosomes to the lysosome, containing a mixture of newly synthesized lysosomal proteins, i.e. p67 and trypanopain, and endocytosed nutrients, i.e. LDL particles and transferrin. 9. CopII vesicles from the ER to the Golgi, carrying all newly synthesized proteins destined for the endocytic pathway. 10. Putative non-CopII vesicles going to and from the ER and the Golgi, possibly carrying lipids, e.g. ceramide and P*C*. 11. Putative SM-enriched vesicle trafficking of VSG directly from the Golgi to the flagellar pocket.

Collectively, these results allow us to propose an updated model of the endocytic and exocytic pathways in bloodstream form *T. brucei* (Fig. 9A), along with an explanation of the effect on these pathways caused by the nSMase cKO grown under permissive conditions (Fig. 9B).

Let us discuss the model in the bloodstream form of the parasite. PC exclusively *de novo* synthesized via the Kennedy pathway (Gibellini *et al*., 2009) and ceramide are utilized by a mitochondrial resident SM synthase to form SM. This SM is then translocated to the ER, via the mitochondria-associated membrane fraction (Bionda *et al*., 2004; Achleitner *et al*., 1999), where the SM encounters the nSMase, forming ceramide and choline–phosphate (Vacaru *et al*., 2009). This ER pool of ceramide now travels with more PC from the ER to the Golgi (Fig. 9, route 9) (Testerink *et al*., 2009), where in the *trans*-Golgi cisterna, a different SM synthase, SLS4 (Tb09211.1000) (Sutterwala *et al*., 2008), utilizes these lipid precursors to reform SM, which is subsequently used for post-Golgi processing of GPI-anchored proteins, i.e VSG. This is similar to the situation in yeast, where IPC, formed in the Golgi from ceramide *de novo* synthesies in the ER and PI, influences GPI-anchored protein trafficking (Watanabe *et al*., 2002). This explains why inhibition of the ceramide *de novo* pathway by myrocin, although lethal did not affect transport of GPI-anchored VSG (Sutterwala *et al*., 2007).

In the case of the nSMase cKO grown under permissive conditions, decreasing levels of nSMase activity directly cause a lack of ceramide and ultimately a lack of PC (discussed eariler) in the ER, which subsequently reduces the amount of ceramide travelling to the Golgi (Fig. 9, route 9). The subsequent lack of SM synthesis in the Golgi decreases the rate (flux) at which SM-enriched vesicles containing newly synthesized VSG can be translocated to the recycling endosomes (Fig. 9, route 6), and beyond to the flagellar pocket (Fig. 9, route 5). This subsequently decreases the flux exiting the late endosomes (Fig. 9, route 4), causing them to enlarge, and reduces the endocytic rate of pre-exisitng cell surface VSG (Fig. 9, route 1), from the early endosomes to the recycling endosomes (Fig. 9, route 2).

As in other organisms a general question arises, why SM is not translocated from the ER to the Golgi? The possible answer may be that transportation of SM from the ER to Golgi is either detrimental for the *cis*-Golgi cisterna and/or the enzymes therein, i.e. glycosidases and glycosyltransferases. Alternatively, SM is not suitable for Cop II vesicle formation (Fig. 9, route 9), which is used for protein trafficking from ER to Golgi (Hughes and Stephens, 2008). Another previously proposed possibility is that lipids such as ceramide and PC are transported to the Golgi via CopII independent vesicles (Funato and Reizman, 2001) (Fig. 9, route 10), but again why SM cannot be translocated using these vesicles is unknown. Interestingly, *T. brucei* has recently been shown to use a subset of Cop II vesicles for selectively transporting GPI-anchored cargo, e.g. VSG, from the ER to Golgi (Sevova and Bangs, 2009), these vesicles may also contain the ceramide and PC for SM synthesis.

The model suggests that post-Golgi sorting of proteins takes place at the *trans*-Golgi network either by clathrin-coated vesicles to the late endosome (Fig. 9, route 7), or SM-enriched vesicles to the recycling endosomes (Fig. 9, route 6).

Proteins such as p67 and trypanopain targeted to be translocated to the lysosome (Fig. 9, route 8) are unaffected by the lack of SM formation in the Golgi in the nSMase cKO cells grown under permissive conditions. Note, processing and sorting in the enlarged late endosome of some proteins are affected, i.e. trypanopain, thus it is likely that p67 processing by trypanopain, normally lysosomal, now partially happens in the enlarged late endosomes.

In the late endosomes lysomal targeted proteins are joined by endocytosed nutrients, i.e. transferrin and LDL, that have previously been sorted from the recycling VSG in the early endosomes (reviewed in Overath and Engstler, 2004) (Fig. 9, route 3), are translocated to the lysosome (Fig. 9, route 8).

Other cell surface proteins, i.e. invariant surface glycoprotein 65 and the recycling transferrin receptor, are sorted to the recycling endosomes (Fig. 9, route 4) to join the recycling VSG and the newly synthesized VSG on route to the flagellar pocket (Engstler *et al*., 2004).

The alternative post-Golgi sorting pathway utilizes SM-enriched vesicles to carry newly synthesized VSG, either via the recycling endosomal network (Fig. 9, route 6) or as has been putatively suggested, directly to the flagellar pocket (Fig. 9, route 11).

Here in the recycling endosomes is the most likely place that myristate exchange of pre-existing and newly synthesized GPI-anchored VSG and potentially other GPI-anchored proteins, i.e. transferrin receptor takes place (Buxbaum *et al*., 1996).

The high flux of SM-enriched recycling endosomes delivers cargo to the flagellar pocket (Fig. 9, route 5), allowing the SM to be recycled via endocytosis (Fig. 9, route 1), via the early and late endosomes to the lysosome (Fig. 9, routes 3 and 8), where it will be catabolized by the putative acidic SMase (Tb 927.4.1120), whose activity has been shown to work on SM and PC containing LDL (Coppens *et al.*, 1995).

Based upon this model and our results, the flux through the endosomal pathway is highly dependent upon the high rates of flux through both avenues of post-Golgi sorting, i.e. the late endosome and the recycling endosomes. To extrapolate this model further, if there is a reduction in the flux from the post-Golgi sorting to the recycling endosomes, and ultimately to the flagellar pocket (exocytosis) (Fig. 9B, routes 6 and 5), as in the nSMase cKO or the VSG RNAi (Sheader *et al.*, 2005; Smith *et al.*, 2009), the flux to and from the late endosome (Fig. 9B, routes 3 and 4) is also affected; in turn causing a decrease in the rate of protein trafficking through the exocytic pathway and ultimately the endocytic pathway. This lack of protein trafficking to the recycling endosomes causes an enlargement of the late endosome. Thus, the new material entering the flagellar pocket cannot be processed leading to an enlargement of the flagellar pocket, now characteristically known as a ‘Big-Eye’ phenotype.

So, the high endocytic and exocytic rates in bloodstream form *T. brucei* are dependent upon high flux of SM-enriched vesicles from the Golgi to the recycling endosomes and beyond to the flagellar pocket/cell surface. This explains the difference in the significantly higher endocytic and exocytic fluxes in the mammalian bloodstream form of the parasite compared with that of the procyclic insect form of the parasites.

In summary, the *T. brucei* nSMase has been recombinatly expressed and shown to be catalytically active. A high-throughput assay has been developed allowing future high-throughput screening. Creation of a conditional knockout in the bloodstream of the parasite allowed genetic validation as a drug target, and the phenotype of which has given new insight into the highly active endocytic and exocytic pathways in bloodstream *T. brucei.*

This work clearly demonstrates that a decrease in the rate of deposition of newly synthesized VSG on the cell surface causes a corresponding decrease in the recycling of VSG, allowing the trypanosome to maintain an optimum cell density of its crucial VSG protective coat. Thus, a new possible strategy in the fight against African trypanosomasis is to find drugs that selectively interfere with the post-Golgi transport of VSG. To this end we are currently investigating nSMase inhibitors as potential anti-trypanosomal drugs.

## Experimental procedures

### Cloning of the *T. brucei* nSMase gene and ligation into pLew vectors

A putative nSMase gene was identified in the *T. brucei* genome database (Tb927.5.3710 – Sanger Institute). The ORF was amplified from *T. brucei* strain 427 genomic DNA using the forward and reverse primers 5′-GAGGAGAAGCTTATGGCTGCAGAGATAACTG-3′ and 5′-TGCTTAATTAATTTACAACCATTACCTTTATGC-3′ containing HindIII and PacI restriction sites respectively (underlined). A band of the expected size of *c*. 1.7 kb was amplified using KOD Hot Start DNA polymerase (Merck Biosciences Ltd)*,* purified using a QIAquick PCR purification kit (Qiagen) and cloned into pCR-Blunt II TOPO (Invitrogen). The *Tb*nSMase ORF was excised using HindIII and PacI, and subsequently ligated into the tetracycline-inducible expression vectors pLew82 and pLew100 (Wirtz *et al*., 1999) via the HindIII and PacI restriction sites. Plasmid DNA was prepared by linearization with NotI subsequently precipitated with sodium acetate/ethanol and redissolved in sterile water to a final concentration of 2 µg µl^−1^, ready for transfection.

### Construction of *T. brucei* gene replacement cassettes

The 5′ and 3′ untranslated regions (UTRs) immediately adjacent to the nSMase ORF were amplified from *T. brucei* genomic DNA using KOD Hot Start DNA polymerase. The primers 5′-ATAAGTAAGCGGCCGCGCGTTTGGGCTGTTCTCGTT-3′ and 5′-*CGTTTAAACTTACGGACCGTCAAGCTT*TTTTGAGTTTAATTCAGTGGG-3′ were used for the 5′ UTR, amplifying the expected 468 bp product, and primers 5′-*tgacggtccgtaagtttaaacggATCC*AGAGGATTGCTTCATCATCATG-3′ and 5′-ATAAGTAAGCGGCCGCTCCCCTCTGCGTAGTGGTAAAATA-3′ resulted in the correct 298 bp product of the 3′ UTR. These amplified products were used in a subsequent knitting PCR, resulting in a 744 bp product in which the 5′ UTR was joined to the 3′ UTR via a short complementary BamHI-HindIII linker region contained within the described primers (italics). This PCR product was ligated into pGEM-5Zf(+) (Promega) via the NotI sites (underlined in the original primers) followed by the insertion of the hygromycin (HYG) or puromycin (PAC) resistance genes between the cut BamHI and HindIII restriction sites. Plasmid DNA was prepared using a QIAprep Miniprep Plasmid Kit (Qiagen), and digested with NotI upon precipitation with sodium acetate/ethanol it was redissolved in sterile water to a final concentration of 2 µg µl^−1^, ready for transfection.

### Cultivation and genetic modification of *T. brucei*

Bloodstream form *T. brucei* strain 427, which had been previously modified to express both T7 polymerase and the tetracycline repressor protein (Wirtz *et al*., 1999), is referred to here as wild-type cells for convenience. Cells were grown in HMI-9 media supplemented with G418 (2.5 µg ml^−1^), at 37°C with 5% CO_2_ as described elsewhere (Wirtz *et al*., 1999; Chang *et al*., 2002; Roper *et al*., 2002). Transformation conditions and subsequent drug selection were also described elsewhere (Burkard *et al*., 2007). Addition of tetracycline to the media when required was at a final concentration of 1 µg ml^−1^. For tetracycline-free experiments, Tet system-approved fetal calf serum (Clontech) was used and cells were washed three times in tetracycline-free HMI-9 and resuspended in the same tetracycline-free media at 5 × 10^4^ cells ml^−1^. Cells were counted each day and were passaged only when the density was greater than 7 × 10^5^ cells ml^−1^ (normally every second day).

### Expression of *T. brucei* nSMase in *E. coli*

The nSMase ORF was amplified from the *Tb*nSMase*-TOPO* plasmid with KOD Hot Start DNA polymerase using the primers 5′-GAGGGATCCATGGCTGCAGAGATAACTGTTC-3′ and 5′-TGCCCCGGGTCATTTACAACCATTACCTTTATG-3′, resulting in a single band of *c*. 1.7 kb. The amplified product was purified using a QIAquick PCR purification kit (Qiagen) prior to blunt-end cloning into pCR-Blunt II-TOPO (Invitrogen) for sequencing. The reading frame was digested out and ligated into the pGEX-6P-1 vector (GE Healthcare) using the BamHI and SmaI sites (underlined) and the sequence confirmed. This construct was transformed into *E. coli* C43 (DE3) competent cells for expression. A single colony was used to inoculate 5 ml of Luria–Bertani media containing 100 µg ml^−1^ ampicillin and 0.5% glucose (w/v) and grown at 37°C overnight. The 5 ml was added to 500 ml of overnight express instant TB medium (Merck Biosciences Ltd) containing 100 µg ml^−1^ ampicillin and grown at 37°C for 4 h, after which the culture was incubated for a further 20 h at 25°C. Cells were harvested by centrifugation (1800 *g*, 15 min) and resuspended in lysis buffer [50 mM Tris pH 8.0, 300 mM NaCl, 10% Glycerol (v/v), 5 mM MgCl_2_, 1 mM DTT] and incubated at 37°C with 0.2 mg ml^−1^ lysozyme (Sigma) and 250 U benzonase nuclease (Merck) for 30 min. Cells were disrupted by sonication (4 × 60 s) on ice and the cell debris, whole cells and ghosts were cleared by slow-speed centrifugation (14 500 *g*, 10 min, 4°C). Expression and identification of the GST–*Tb*nSMase was confirmed by Western blotting using anti-GST antibodies (Covance) and ECL Western detection reagents (Amersham). Subsequently, *E. coli* membranes containing GST–*Tb*nSMase were collected by high-speed centrifugation (100 000 *g*, 1 h), washed in PBS and suspended in 100 mM Tris (pH 7.4), 10 mM MgCl_2_ and 20% glycerol and stored as aliquots at −80°C. Protein was extracted from the GST–*Tb*nSMase membranes by solubilizing in 1% Triton X-100 prior to determining the total protein content using the BCA protein assay kit (Thermo Scientific).

### nSMase enzyme assays

Sphingomyelinase activity was confirmed by the use of radioactive [^3^H]-SM, washed membranes (containing 127 µg of total protein as determined by using a BCA assay kit (Thermo Scientific) encoding GST–*Tb*nSMase incubated in 100 mM Bis-Tris propane (pH 7.5), differing concentrations of MgCl_2_ (as stated in the individual figures), 0.5% glycerol with 25 nCi [choline-methyl-^3^H]SM (50 Ci mmol^−1^, ARC) and 50 pmol cold SM presented as mixed micelles with 0.1% Triton-X 100. Reactions were incubated at 37°C for 120 min. Hydrolysis of PC was assayed in an identical manner with 30 nCi [^3^H-choline-methyl]PC (60 Ci mmol^−1^, ARC) and 50 pmol cold PC presented as mixed micelles with 0.1% Triton-X 100. For analysis of the optimum pH for activity, the pH of the reaction buffer was altered accordingly. Reactions (50 µl) were stopped by the addition of 800 µl chloroform:methanol (2:1 v/v) followed by 250 µl H_2_O. After phase separation, aqueous and organic phases were dried down and resuspended in 50% propan-1-ol or 100% butan-1-ol and examined by TLC using solvent systems of methanol:0.6% NaCl:25% NH_3_ (100:100:10) or chloroform:methanol:water (10:10:3 v/v) respectively.

Neutral sphingomyelinase activity was further characterized using the Amplex Red sphingomyelinase assay kit (Invitrogen Ltd, He *et al*., 2002). Briefly, washed membranes (containing 254 µg of total protein) encoding GST–*Tb*nSMase were incubated in 100 mM Tris-HCl (pH 7.4), 10 mM MgCl_2_, 0.5% glycerol with varying amounts of SM (presented as mixed micelles with 0.1% Triton-X 100). Production of choline phosphate from SM was detected by inclusion of the standard Amplex red assay components in a final assay volume of 200 µl. Reaction mixtures were incubated at 30°C for 1 h and the fluorescence product generated measured kinetically (Excitation 56 0nm, Emission 587 nm). *T. brucei* bloodstream form washed membranes prepared as above and containing an estimated 35 µg of total protein were assayed for nSMase activity in an identical manner with 100 µM SM (presented as mixed micelles with 0.1% Triton-X 100). Inhibitor studies with the trypanosomal membranes were conducted by pre-incubation of up to 500 µM of inhibitor for 15 min, prior to the addition of 100 µM SM, and Amplex red for 30 min 37°C.

Inhibitor studies with GST–*Tb*nSMase were conducted by pre-incubation of 50 µM inhibitors for 30 min prior to the addition of 100 µM SM and Amplex red for 30 min 37°C.

### Southern blots

The nSMase ORF was PCR-amplified using the same ORF primers as for the pLew vectors and gel purified and labelled with DIG- (Roche). Genomic *T. brucei* DNA (3 µg) was digested with various restriction enzymes. Digestion products were separated on a 0.8% agarose gel and transferred to Hybond-N+ membrane (Amersham), and hybridized overnight in ULTRA-HYB (Ambion) at 42°C with the dig-labelled nSMase ORF probe. Stringency washes were carried out at 42°C, and consisted of two washes at low stringency for 15 min each [2× SSC (300 mM NaCl, 30 mM sodium citrate), 0.1% SDS] and two washes at high stringency again for 15 min each [0.2× SSC (30 mM NaCl, 3 mM sodium citrate), 0.1% SDS]. Bound probe was detected by anti-Dig antibody following manufacturers instructions.

### RNA isolation and cDNA synthesis

Total RNA was isolated from bloodstream form *T. brucei* using the RNeasy mini kit (Qiagen). *Tb*nSMase-specific cDNA was generated and amplified using the specific forward 5′-GAGGAGCATATGGCTGCAGAGATAACTGTTC-3′ and reverse 5′-TGCGGATCCTCAAGCTTCACATAGAGACGCCTC-3′ primers using the SuperScript III One step RT-PCR kit with Platinum Taq (Invitrogen). As a negative control to exclude DNA contamination of the RNA sample, reverse transcriptase was omitted from the reaction and replaced with GoTaq polymerase (Promega). Inositol-3-phosphate synthase (*TbINO1*) cDNA was also amplified using the forward and reverse primers 5′-AGCGGCAAGCTTCTATGCCAGCCGTCCGTACGAAAA-3′ and 5′-CGACCTCGAGGTCAACTTCCCACGCCGCGAAGGAAAGGCAG-3′ to confirm equal RNA loading. The PCR products were then run on a 1% agarose gel.

### Subcellular localization studies

Tetracycline-induced mid-log *T. brucei* nSMase*-HA*^Ti^ bloodstream form cells were harvested by centrifugation (800 *g*, 10 min) and used for either immunofluorescence or differential centrifugation as previously described (Martin and Smith, 2006b). The blocked cells were incubated with either rat α-HA (Roche) or rabbit α-trypanopain antibody (James Bangs, University of Wisconsin-Madison) followed by their respective secondary; FITC conjugated rabbit anti-rat (DakoCytomation) and TRITC conjugated Goat anti-rabbit immunoglobulins (Sigma). 4,6-diamidino-2-phenylindole (DAPI) staining was used to reveal the localization of the nuclear and kDNA.

Differential centrifugation and Western analysis for HA-tagged protein were detected using the primary rat α-HA (Roche), and secondary HRP-conjugated rabbit α-rat (Jackson) antibodies and ECL Western detection reagents (Amersham) were used as described previously (Martin and Smith, 2006a).

### *In vivo T. brucei* metabolic labelling

For each metabolic labelling, mid-log trypanosomes were centrifuged (800 *g*, 10 min), washed in minimal essential media before being resuspended in the specific supplement free media at a final concentration of 1 × 10^7^ cells ml^−1^. Cells were labelled for 2 h at 37°C with [9,10-^3^H] myristic acid (47 Ci mmol^−1^, Perkin Elmer) complexed to defatted-BSA (1 mol mol^−1^) in a shaking water bath. Cells were collected by centrifugation (800 *g*, 10 min), washed twice in trypanosome dilution buffer (TDB) buffer and processed for either lipid or protein analysis. Lipids were extracted using chloroform: methanol: water (10:10:3 v/v) for 1 h, the supernatant removed and the pellet re-extracted with chloroform:methanol (2:1 v/v) for 1 h. The pooled supernatants were dried, prior to desalting using butanol/water partioning. Lipids were separated on silica 60 high-performance thin layer chromatography (HPTLC) plates, with chloroform : methanol : ammonia (13 M) : ammonium acetate (1 M) : water, (180 : 140 : 9 : 9 : 23 v/v) as the solvent. Radiolabelled lipids were detected by fluorography at −70°C, after spraying with En^3^hance and using Kodak XAR-5 film with an intensifying screen. Protein samples (cell pellets) were quenched in 2× SDS-PAGE sample buffer and heated at 95°C for 5 min. Proteins were separated on a 10% SDS-PAGE gel and visualized by Coomassie blue staining. To detect [^3^H]-labelled proteins the destained gel was soaked in En^3^hance (NEN) for 30 min, washed with water three times, soaked in 10% glycerol and dried and exposed to XAR-5 film at −80°C.

When labelling with [^35^S]-methionine, mid-log cells were collected by centrifugation, washed in methionine free minimal essential media and resuspended in the same media at a final concentration of 1 × 10^7^cells ml^−1^. Cells were labelled for 1 h with 20 µCi ml^−1^[^35^S]-methionine (MP Biomedicals, 1175 Ci mmol^−1^) at 37°C. Small-scale sVSG purification was as performed as described previously (Jones *et al*., 2005).

For the immunoprecipitation (IP) of [^35^S]-p67 from *T. brucei*, the protocol of Alexander *et al*. (2002) was followed with some modifications. Briefly, 5 × 10^6^[^35^S]-methionine pulse-labelled (1 h) trypanosomes were harvested at each time point (0 and 2 h), washed once in PBS and solubilized in 500 µl of lysis buffer [TEN buffer, 1% Nonidet P-40, 0.5% deoxycholate, 0.1% SDS (TEN buffer = 50 mM Tris-HCl, pH 7.5, 150 mM NaCl, 5 mM EDTA)] containing a protease inhibitor cocktail. The extracts were precleared by centrifugation (14 000 rpm, 5 min) before detergents (1% Nonidet P-40, 0.5% deoxycholate, 0.1% SDS) were added to the culture supernatants to the same concentration as the lysis buffer (1% Nonidet P-40, 0.5% deoxycholate, 0.1% SDS). Monoclonal anti-p67 (mAb280) antibody (a gift of James Bangs, University of Wisconsin-Madison), which recognizes an uncharacterized peptide epitope in the p67 lumenal domain, was preadsorbed to Protein G-Sepharose 4B Fast Flow resin (Sigma) prior to incubation with the solubilized protein for 1 h at 4°C. After incubation, resin beads were washed four times with lysis buffer and once with TEN buffer. Following centrifugation the resin from each time point was suspended in an equal volume of 2× SDS-PAGE sample buffer prior to denaturing at 100°C for 10 min, to release the complexes from the protein G sepharose. Proteins were separated on a 10% SDS-PAGE gel and visualized by Coomassie blue staining. To detect [^35^S]-labelled proteins the destained gel was soaked in En^3^hance (NEN) and detected by autoradiography as described before.

### FACS analysis

Cells were stained with propidium iodide as described previously (Hammarton *et al*., 2003). Briefly, cells were fixed in 70% methanol, 30% PBS (1×10^6^ cells ml^−1^) and incubated at 4°C overnight. Cells were washed in cold PBS, resuspended in 1 ml of PBS containing 10 µg ml^−1^ propidium iodide and 10 µg ml^−1^ RNase A, and incubated at 37°C for 45 min. FACS was performed using a Becton Dickinson FACScan using detector FL2-A.

Duplicate data sets were collected and analysed using WinMDI software (http://facs.scripps.edu) utilizing 10 000 events which were collected for each sample. Cells in a particular stage of the cell cycle (G0/G1, S, G2/M or >G2/M) are indicated as a percentage of the total. The gates used for quantification of the different stages are as in Sheader *et al*. (2005).

### Lipidomic analysis

Phospholipid analysis was performed on bloodstream wild-type and *Tb*nSMase cKO trypanosomes (1 × 10^8^) in the presence or absence of tetracycline for 42 h. Cells were harvested and washed in TDB and extracted according to the method of Bligh–Dyer (Bligh and Dyer, 1959), were dried under N_2_, and were stored at 4°C until analysed by electrospray mass spectrometry (ES-MS and ES-MS/MS) as described previously (Richmond and Smith, 2007; Gibellini *et al*., 2009; Richmond *et al*., 2010).

### Western blotting

Specific *T. brucei* proteins were detected by Western blot analysis, using either rabbit α-VSG221 or rabbit α-trypanopain (gifts from James Bangs, University of Wisconsin-Madison) or rabbit α-PDI-2 (a gift from Derek Nolan, Trinity College Dublin) antibodies with secondary rat α-rabbit HRP-conjugated antibody (Jackson ImmunoResearch Ltd). ECL detection reagents (GE Healthcare) were utilized to visualize the signal.

### FITC-BSA uptake assay

Mid-log trypanosomes were harvested by centrifugation (800 *g*, 10 min) and resuspended at 1 × 10^6^ ml^−1^ in minimal essential media. The cells are preincubated for 10 min at 37°C before FITC-BSA is added at a final concentration of 5 mg ml^−1^. After incubation for 30 min, the cells were harvested by centrifugation and washed twice in PBS to remove unbound FITC-BSA, prior to fixation in 4% paraformaldehyde and processed for immunofluorescence analysis as described above.

### Electron Microscopy

For transmission electron microscopy cell pellets were fixed for up to 24 h with Peter's fixative (1.25% glutataldehyde, 1% paraformaldehyde in 0.08 M sodium cacodylate pH 7.2 with 0.02% calcium chloride). Samples were rinsed twice in 1% tannic acid for 10 min post-fixed/stained with 0.5% osmium tetraoxide (aq) for 1 h. Specimens were then rinsed twice in distilled water before fixation/staining in 1% uranyl acid followed by infiltration/embedding in Spurr resin. Sections were cut using a Leica Ultracut UCT microtome, mounted on pioloform coated copper grids, post-stained with 3% uranyl acid (aq) and Reynold's lead citrate and examined using a Tecnai 12 BioTWIN made by FEI, and it was operated at 120 kV. Images were collected using an SIS Megaview III, as 2 × 2 montages.
