# Supplementary material for: The essential neutral sphingomyelinase is involved in the trafficking of the variant surface glycoprotein in the bloodstream form of Trypanosoma brucei
Source: Mol Microbiol. 2010 Apr 15;76(6):1461–82. doi: 10.1111/j.1365-2958.2010.07151.x (PMC2904498; doi:10.1111/j.1365-2958.2010.07151.x)

***SUPPLEMENTARY DATA***

| Peak | m/z[a] | *HR m/z*[b] | Lipid[c] | Principal component[d,] | *Theor m/z*[e] |
|---|---|---|---|---|---|
| 1 | 520 | 520.30 | 18:2 | 0:0/18:2 | 520.3398 |
| 2 | 522 | 522.31 | 18:1 | 0:0/18:1 | 522.3554 |
| 3 | 524 | 524.33 | 18:0 | 0:0/18:0 | 524.3711 |
| 4 | 544 | 544.34 | 20:4 | 0:0/20:4 | 544.3398 |
| 5 | 546 | 546.32 | 20:3 | 0:0/20:3 | 546.3554 |
| 6 | 548 | 548.37 | 20:2 | 0:0/20:2 | 548.3711 |
| 7 | 568 | 568.35 | 22:6 | 0:0/22:6 | 568.3398 |
| 8 | 570 | 570.37 | 22:5 | 0:0/22:5 | 570.3554 |
| 9 | 572 | 572.37 | 22:4 | 0:0/22:4 | 572.3711 |
| 10 | 646 | 646.45 | 26:2 | 0:0/26:2 | 646.4443 |
| 11 | 648 | 648.47 | 26:1 | 0:0/26:1 | 648.4599 |
| 12 | 650 | 650.49 | 26:0 | 0:0/26:0 | 650.4756 |
| 13 | 660 | 660.59 | a-28:2 | a-14:0/14:2 | 660.5363 |
| 14 | 662 | 663.55 | a-28:1 | a-14:0/14:1 | 662.5119 |
| 15 | 664 | 664.51 | a-28:0 | a-14:0/14:0 | 664.5276 |
| 16 | 674 | 674,49 | 28:2 | 14:0/14:2 | 674.4756 |
| 17 | 676 | 676.52 | 28:1 | 14:0/14:2 | 676.4912 |
| 18 | 678 | 878.53 | 28:0 | 14:0/14:0 | 678.5069 |
| 19 | 675 | 675.52 | SM(14:0) | Cer 18:1/14:0 | 675.54 |
| 20 | 686 | 686.32 | e-30:2 | e-14:1/16:2 | 686.5220 |
| 21 | 688 | 686.29 | e-30:1, a-30:2 | a-14:0/16:2 | 688.5376 |
| 22 | 690 | 690.56 | e-30:0, a-30:1 | a-14:0/16:1 | 690.5432 |
| 23 | 692 | 692.53 | a-30:0 | a-14:0/16:0 | 692.5589 |
| 24 | 698 | 698.49 | 30:4 | 16:2/14:2 | 698.4756 |
| 25 | 700 | 700.52 | 30:3 | 16:2/14:1 | 700.4912 |
| 26 | 702 | 702.54 | 30:2 | 16:0/14:2 | 702.5069 |
| 27 | 704 | 703.62 | SM (16:0) | Cer 18:1/16:0 | 703.58 |
| 28 | 704 | 704.55 | 30:1 | 16:0/14:1 | 704.5225 |
| 29 | 706 | 706.54 | 30:0 | 16:0/14:0 | 706.5382 |
| 30 | 716 | 716.57 | e-32:1, a-32:2 | e-16:1/16:1 | 716.5589 |
| 31 | 718 | 718.59 | e-32:0, a-32:1 | e-16:1/16:0 | 718.5745 |
| 32 | 720 | 720.62 | a-32:0 | a-16:0/16:0 | 720.5902 |
| 33 | 728 | 728.54 | 32:3 | 16:1/16:2 | 728.5225 |
| 34 | 730 | 730.55 | 32:2 | 16:0/16:2 | 730.5382 |
| 35 | 732 | 732.57 | 32:1 | 16:0/16:1 | 732.5538 |
| 36 | 732 | 731.64 | SM(18:0) | Cer 18:1/18:0 | 731.61 |
| 37 | 734 | 734.58 | 32:0 | 16:0/16:0 | 734.5695 |
| 38 | 738 | 738.58 | e-34:4, a-34:5 | a-14:0/20:5 | 738.5433 |
| 39 | 740 | 740.57 | e-34:3, a-34:4 | a-14:0/20:4 | 740.5589 |
| 40 | 742 | 742.59 | e-34:2, a-34:3 | a-14:0/20:3 | 742.5745 |
| 41 | 744 | 744.63 | e-34:1, a-34:2 | a-16:0/18:2 | 744.5902 |
| 42 | 746 | 746.63 | e-34:0, a-34:1 | a-16:0/18:1 | 746.6058 |
| 43 | 748 | 748.61 | a-34:0 | a-16:0/16:0 | 748.6215 |
| 44 | 754 | 754.55 | 34:4 | 16:2/18:2 | 754.5382 |
| 45 | 756 | 756.57 | 34:3 | 16:1/18:2 | 756.5538 |
| 46 | 758 | 758.59 | 34:2 | 16:0/18:2 | 758.5695 |

| | | | | | |
|---|---|---|---|---|---|
| 47 | 760 | 759.62 | SM(20:0) | Cer18:1/20:0 | 759.64 |
| 48 | 760 | 760.59 | 34:1 | 16:0/18:1 | 760.5851 |
| 49 | 762 | 762.62 | 34:0 | 16:0/18:0 | 762.6008 |
| 50 | 766 | 766.55 | e-36:4, a-36:5 | e-16:1/20:4 | 766.5745 |
| 51 | 768 | 768.55 | e-36:3, a-36:4 | a-16:0/20:4 | 768.5902 |
| 52 | **770** | **770.62** | **e-36:2, a-36:3** | **e-18:1/18:2** | **770.6058** |
| 53 | **772** | **772.65** | **e-36:1, a-36:2** | **a-18:0/18:2** | **772.6215** |
| 54 | **774** | **774.63** | **e-36:0, a-36:1** | **a-18:0/18:1** | **774.6371** |
| 55 | 776 | 776.63 | a-36:0 | a-18:0/18:0 | 776.6528 |
| 56 | 778 | 778.55 | 36:6 | 16:2/20:4 | 778.5382 |
| 57 | 780 | 780.53 | 36:5 | 16:1/20:4 | 780.5538 |
| 58 | 782 | 782.53 | 36:4 | 16:0/20:4 | 782.5695 |
| **59** | **784** | **784.56** | **36:3** | **18:1/18:2** | **784.5851** |
| **60** | **786** | **786.59** | **36:2** | **18:0/18:2 & 18:1/18:1** | **786.6008** |
| **61** | **788** | **788.57** | **36:1** | **18:0/18:1** | **788.6164** |
| **62** | **790** | **789.65** | **SM(22:0)** | **Cer18:0/22:0** | **789.68** |
| 63 | 790 | 790.54 | e-38:6 | e-16:1/22:6 | 790.5745 |
| 64 | 790 | 790.61 | 36:0 | 18:0/18:0 | 790.6321 |
| 65 | 792 | 792.57 | e-38:5, a-36:6 | a-16:0/22:6 | 792.5902 |
| 66 | 794 | 794.54 | e-38:4, a-38:5 | e-18:1/20:4 | 794.5695 |
| 67 | 796 | 796.84 | e-38:3, a-38:4 | a-18:0/20:4 | 796.6215 |
| 68 | 798 | 798.64 | e-38:2, a-38:3 | e-18:1/20:2 | 798.6371 |
| 69 | 800 | 800.68 | e-38:1, a-38:2 | a-18:0/20:2 | 800.6528 |
| 70 | 802 | 802.68 | e-38:0, a-38:1 | a-18:0/20:1 | 802.6684 |
| 71 | 804 | 804.54 | 38:7 | 16:1/22:6 | 804.5538 |
| 72 | 804 | 804.69 | a-38:0 | a-18:0/20:0 | 804.6841 |
| **73** | **806** | **806.53** | **38:6** | **16:0/22:6** | **806.5695** |
| **74** | **808** | **808.53** | **38:5** | **16:0/22:5** | **808.5851** |
| 75 | **810** | **810.58** | **38:4** | **18:0/20:4 & 16:0/22:4** | **810.6008** |
| 76 | **812** | **812.60** | **38:3** | **18:0/20:3** | **812.6164** |
| 77 | **814** | **814.62** | **38:2** | **18:0/20:2** | **814.6321** |
| 78 | 816 | 816.63 | 38:1 | 18:0/20:1 | 816.6477 |
| 79 | 818 | 818.57 | e-40:6 | e-18:1/22:6 | 818.6058 |
| 80 | 818 | 818.67 | 38:0 | 18:0/20:0 | 818.6634 |
| 81 | 820 | 820.61 | e-40:5, a-40:6 | a-18:0/22:6 | 820.6215 |
| 82 | 822 | 822.61 | e-40:4, a-40:5 | e-18:1/22:4 | 822.6371 |
| 83 | 824 | 824.63 | e-40:3, a-40:4 | a-18:0/22:4 | 824.6528 |
| 84 | 826 | 826.61 | e-40:2, a-40:3 | e-18:1/22:2 | 826.6321 |
| 85 | 828 | 828.68 | e-40:1, a-40:2 | a-18:0/22:2 | 828.6841 |
| 86 | 830 | 830.68 | e-40:0, a-40:1 | e-18:1/22:0 | 830.6997 |
| 87 | 832 | 832.70 | a-40:0 | a-18:0/22:0 | 832.7154 |
| **88** | **830** | **830.55** | **40:8** | **18:2/22:6** | **830.5695** |
| **89** | **832** | **832.57** | **40:7** | **18:1/22:6** | **832.5851** |
| **90** | **834** | **834.59** | **40:6** | **18:0/22:6** | **834.6008** |
| **91** | **836** | **836.59** | **40:5** | **18:0/22:5** | **836.6164** |
| **92** | **838** | **838.62** | **40:4** | **18:2/22:4** | **838.6321** |
| **93** | **840** | **840.65** | **40:3** | **18:1/22:2** | **840.6477** |
| **94** | **842** | **842.68** | **40:2** | **18:0/22:2** | **842.6634** |
| 95 | 844 | 844.72 | 40:1 | 18:1/22:0 | 844.6790 |
| 96 | 846 | 846.73 | 40:0 | 18:0/22:0 | 846.6947 |

| | | | | | |
|---|---|---|---|---|---|
| 97 | 848 | 848.66 | e-42:6 | e-18:1/24:6 | 848.6528 |
| 98 | 850 | 850.66 | e-42:5 | e-18:1/24:4 | 850.6684 |
| 99 | 852 | 852.68 | a-42:4 | a-18:0/24:4 | 852.6841 |
| 101 | 852 | 852.59 | 42:11 | 20:4/22:5 | 852.5538 |
| 102 | 854 | 854.72 | a-42:3 | e-18:1/24:2 | 854.6997 |
| 103 | 854 | 854.57 | 42:10 | 20:4/22:6 | 854.5695 |
| 104 | 856 | 856.73 | a-42:2 | a-18:0/24:2 | 856.7154 |
| 105 | 856 | 856.59 | 42:9 | 20:4/22:5 | 856.5851 |
| 106 | 858 | 858.75 | a-42:1 | a-18:1/24:0 | 858.7310 |
| 107 | 859 | 858.59 | 42:8 | 18:2/24:6 | 858.6008 |
| 108 | 861 | 860.57 | 42:7 | 18:1/24:6 | 860.6164 |
| 109 | 860 | 860.72 | a-42:0 | a-18:0/24:0 | 860.7467 |
| 110 | 862 | 862.61 | 42:6 | 18:0/24:6 | 862.6321 |
| 111 | 864 | 864.63 | 42:5 | 18:0/24:5 | 864.6477 |
| 112 | 866 | 866.66 | 42:4 | 18:2/24:4 | 866.6634 |
| 113 | 868 | 868.69 | 42:3 | 18:1/24:2 | 868.6790 |
| 114 | 870 | 870.73 | 42:2 | 18:0/24:2 | 870.6947 |
| 115 | 872 | 872.73 | 42:1 | 18:1/24:0 | 872.7103 |
| 116 | 874 | 874.75 | 42:0 | 18:0/24:0 | 874.7260 |
| 117 | 878 | 878.59 | 44:12 | 20:6/24:6 | 878.5695 |
| 118 | 880 | 852.58 | 44:11 | 20:6/24:5 | 852.5565 |
| 119 | 882 | 882.59 | 44:10 | 20:4/24:6 | 882.6008 |
| 120 | 886 | 886.61 | 44:8 | 20:4/24:4 | 886.6321 |
| 121 | 888 | 888.61 | 44:7 | 20:2/24:5 | 888.6477 |
| 122 | 890 | 890.62 | 44:6 | 20:1/24:6 | 890.6634 |
| 123 | 892 | 892.63 | 44:5 | 20:0/24:5 | 892.6790 |
| 124 | 894 | 894.65 | 44:4 | 18:0/26:4 | 894.6947 |
| 125 | 896 | 896.68 | 44:3 | 20:1/24:2 | 896.7103 |
| 126 | 898 | 898.70 | 44:2 | 20:0/24:2 | 898.7260 |
| 127 | 900 | 900.72 | 44:1 | 18:0/26:1 | 900.7416 |
| 128 | 902 | 902.73 | 44:0 | 18:0/26:0 | 902.7573 |

Table S1: Mass spectrometry analysis of PC and sphingomyelin (SM) molecular species in the bloodstream form of *T.brucei*

[a] $[M+H]^+$ ions over charge.
[b] HR $[M+H]^+$ ions over charge (high resolution survey scan) ±0.05m/z
[c] Peak identities refer to total number of carbon atoms and double bonds. The peaks containing the most abundant PC/SM species are in bold.
[d] Defined here as the $[M+H]^+$ ion within the series with the greatest intensity. Precise fatty acyl constituents and their positions are not discernable in positive ion mode; however, where possible, the most likely fatty acid candidates (in parentheses) for the *sn*-1 and *sn*-2 constituents of the molecular species presented were deduced from the available literature.
[e] All of the molecular species detected within the area of the peak are contained within the lowest and highest mass range outliers listed, the degree of unsaturation decreases by one from the lowest mass in each series until the highest mass is met. e = plasmenyl (alkenylacyl); a = plasmanyl (alkylacyl)

***SUPPLEMENATARY FIGURE LEGENDS***

Figure S1; (A) ClustalW alignment of the predicted amino acid sequences of the *T. brucei* nSMase with nSMases from other eukaryotes: *Leishmania major* (XP 001681949), *Trypanosoma cruzi* (XP 814233), *Homo sapiens* (CAA10995), *Saccharomyces cerevisiae* (NP 010935) and *Apis mellifera* (XP 395633). Identical residues are shown in reverse type with black background, conserved residues are shown in reverse type with grey background. Residues required for catalytic activity, substrate binding, and $Mg^{2+}$ binding, in other homologues are indicated by asterisks. The two putative transmembrane helices are underlined. The Endonuclease/Exonuclease/phosphatase Pfam motif PF03372 covers residues 5 – 364.
(B) Phylogenetic analysis using ClustalW of the nSMase homologues from Figure 1A.

Figure S2; Activity of recombinant *Tb*nSMase
Enzyme activity of *Tb*nSMase activity in washed *E.coli* membranes expressing either GST-*Tb*nSMase (1) or N-terminal-$His_6$-*Tb*nSMase (2) or empty vector (3).

Figure S3; Further examples of ER localisation of *Tb*nSMase-$HA^{Ti}$ in bloodstream form *T. brucei*. Fixed *Tb*nSMase-$HA^{Ti}$ cells were incubated with rat anti-HA antibody, and rabbit anti-rat FITC conjugated antibody and DNA stained with DAPI.

FIGURE S4; RT-PCR analysis: Total RNA from procyclic cells (Lane 2), bloodstream form cells (Lane 3). The RT-PCR was performed with *TbnSMase* orf primers, as detailed in *Experimental procedures.*

Figure S5: Lipids were extracted from *T. brucei* wild type (A) and *Tb nSMase* conditional knockout without tetracycline for 42h (B) and analysed by ES-MS-MS for choline-phosphate containing phospholipids by collision induced fragmentation in positive ion mode of parents of 184m/z in wild type cells (A) and *Tb nSMase* conditional knockout without tetracycline for 42h (B).

Figure S6: Lipids were extracted from *Tb nSMase* conditional knockout without tetracycline for 42h and analysed by negative ion mode ES-MS (A) and ES-MS-MS by collision induced fragmentation in negative ion mode of parents of 241m/z for PI species (B), parents of 196m/z for PE species (C) and neutral loss of 87m/z for PS species (D) as described in *Experimental procedures*.

Figure S7: ES-MS-MS daughter ion spectrum of the m/z 771 ion (A), m/z 670 ion (B) and collision induced fragmentation in negative ion mode of parents of 241m/z for Ino-phosphate containing species from the lipid extract of the P100 fraction of *Tb nSMase* conditional knockout without tetracycline for 42h as described in *Experimental procedures*.

Figure S8: Further examples of TEM of wild-type bloodstream form *T,brucei* (A-D) and *TbnSMase* cKO (-tet) showing a big-eye phenotype. . FL, flagellum; FP, flagellar pocket; K, kinetoplast; M, mitochondria: G, glycosome; *, enlarged concentric membrane structure, may be part of the endosomal network.

Figure S9: (A) Coomassie stain protein gel of the IP [$^{35}$S]methionine labelled P67 for Figure 7A, Wild type (lanes 2 and 3) and *TbnSMase* cKO cells grown in the absence of tetracycline for 42 hours (4 and 5). Lanes 6 and 7 show the coomassie blue stain of the total protein [$^{35}$S]-methionine labelling with the corresponding autoradiograph (lanes 8 and 9). (B) Anti-VSG Western of VSG in total lysate (lanes 1 and 2) and sVSG preparations (lanes 3 and 4) from wild type (lanes 1 and 3) and *TbnSMase* cKO cells grown in the absence of tetracycline for 42 hours (2 and 4).

Figure S10; Further examples of the morphological "Big-Eye" phenotype of the *Tb*nSMase conditional knockout grown in the absence of tetracycline for 42 hours are shown as merged fluorescence-phase images of DAPI and anti-trypanopain.

Figure S11; Anti-PDI-2 western of wild type (lane 1) and *TbnSMase* cKO cells grown in the absence of tetracycline for 42 hours (lane 2).

# Figure S1

A

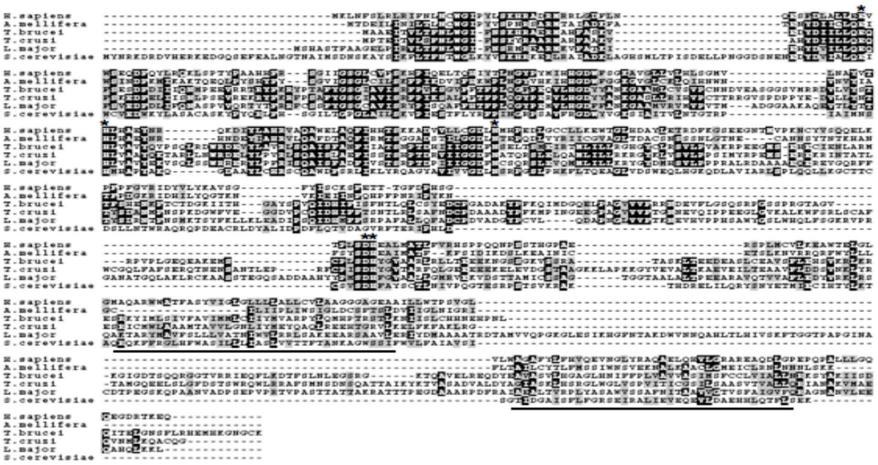

B

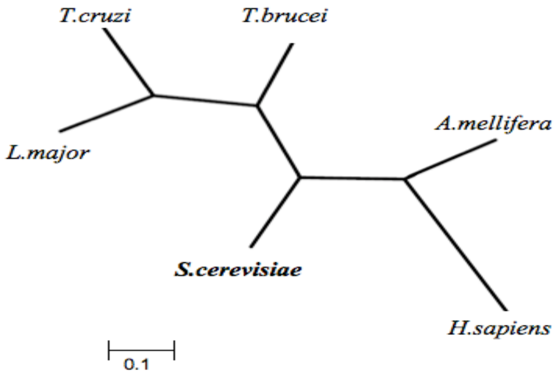
T.cruzi
T.brucei
L.major
A.mellifera
S.cerevisiae
H.sapiens
0.1

# Figure S2

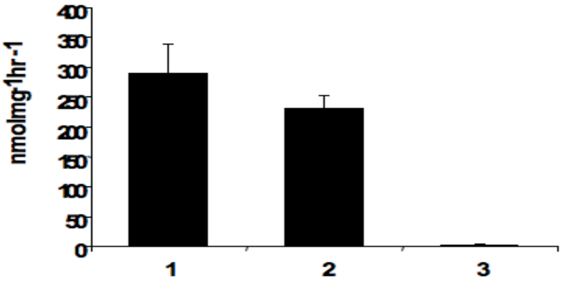

Figure S3

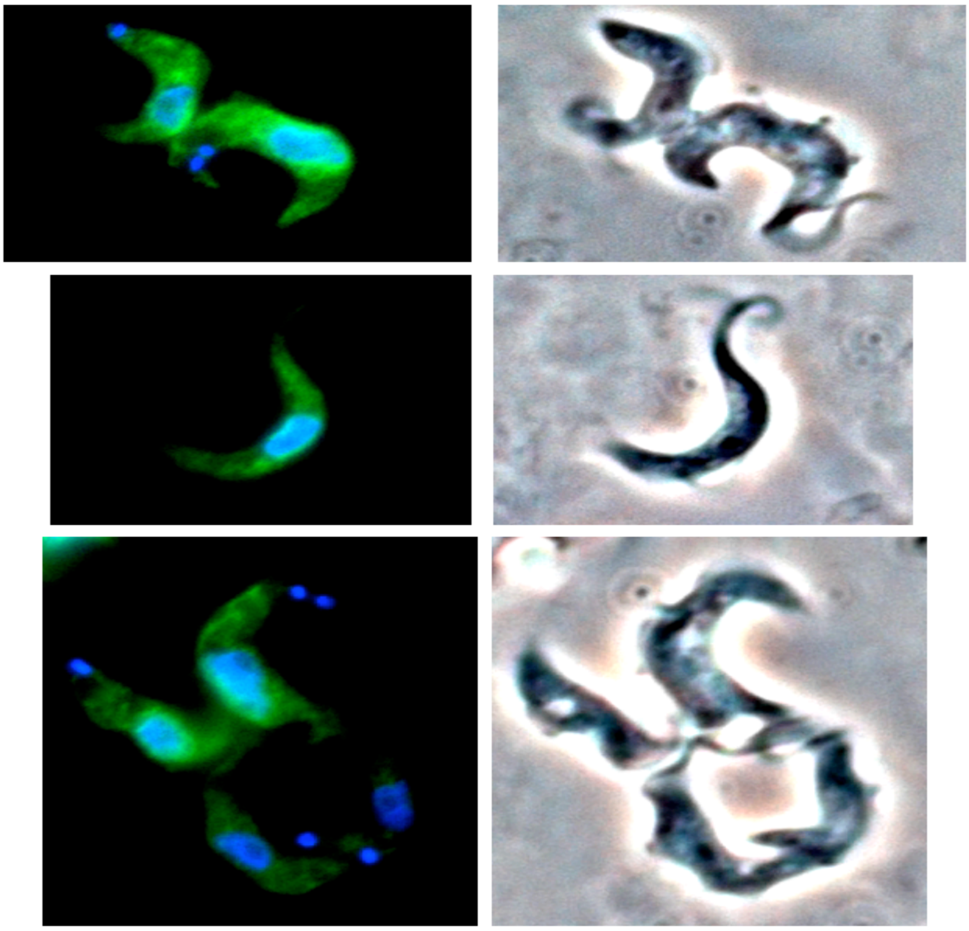

Figure S4

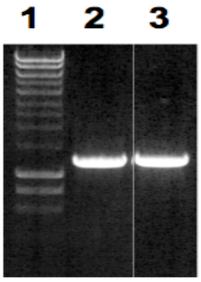

# Figure S5

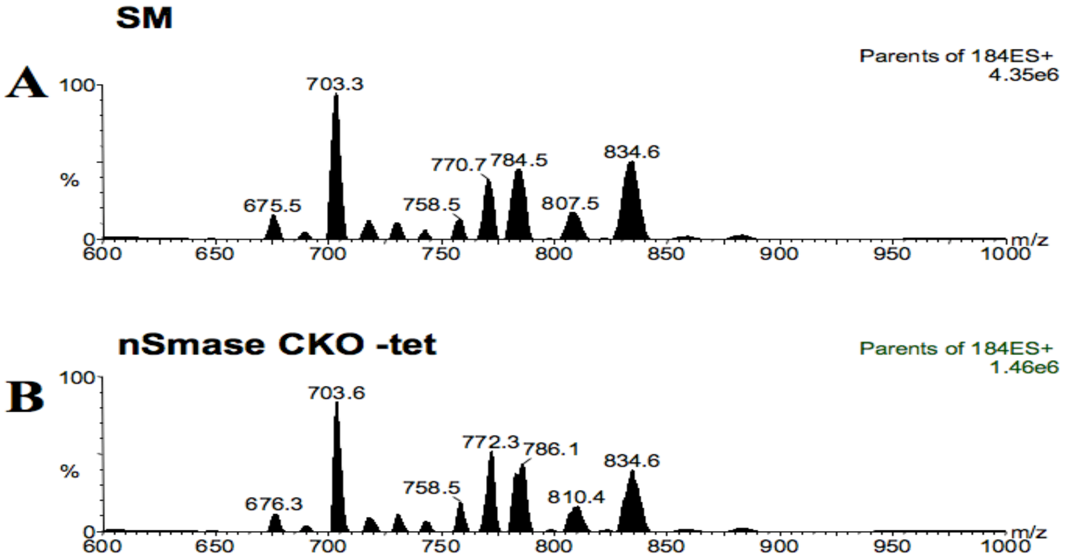
SM
A
Parents of 184ES+
4.35e6
100
%
0
703.3
675.5
758.5
770.7
784.5
807.5
834.6
600
650
700
750
800
850
900
950
1000
m/z
nSmase CKO -tet
B
Parents of 184ES+
1.46e6
100
%
0
703.6
676.3
758.5
772.3
786.1
810.4
834.6
600
650
700
750
800
850
900
950
1000
m/z

Figure S6

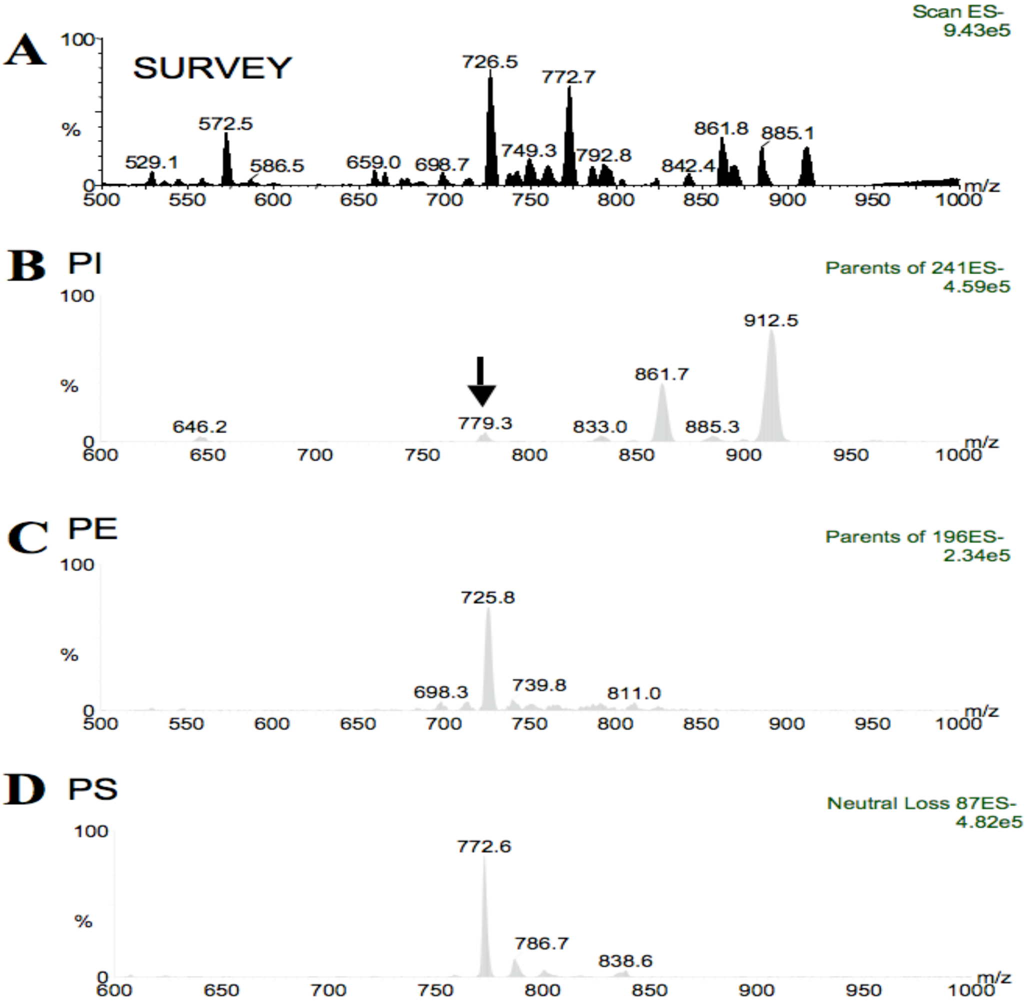
A
SURVEY
Scan ES-
9.43e5
726.5
772.7
572.5
861.8
885.1
749.3
792.8
529.1
586.5
659.0
698.7
842.4
B
PI
Parents of 241ES-
4.59e5
912.5
861.7
646.2
779.3
833.0
885.3
C
PE
Parents of 196ES-
2.34e5
725.8
698.3
739.8
811.0
D
PS
Neutral Loss 87ES-
4.82e5
772.6
786.7
838.6
%
m/z

Figure S7

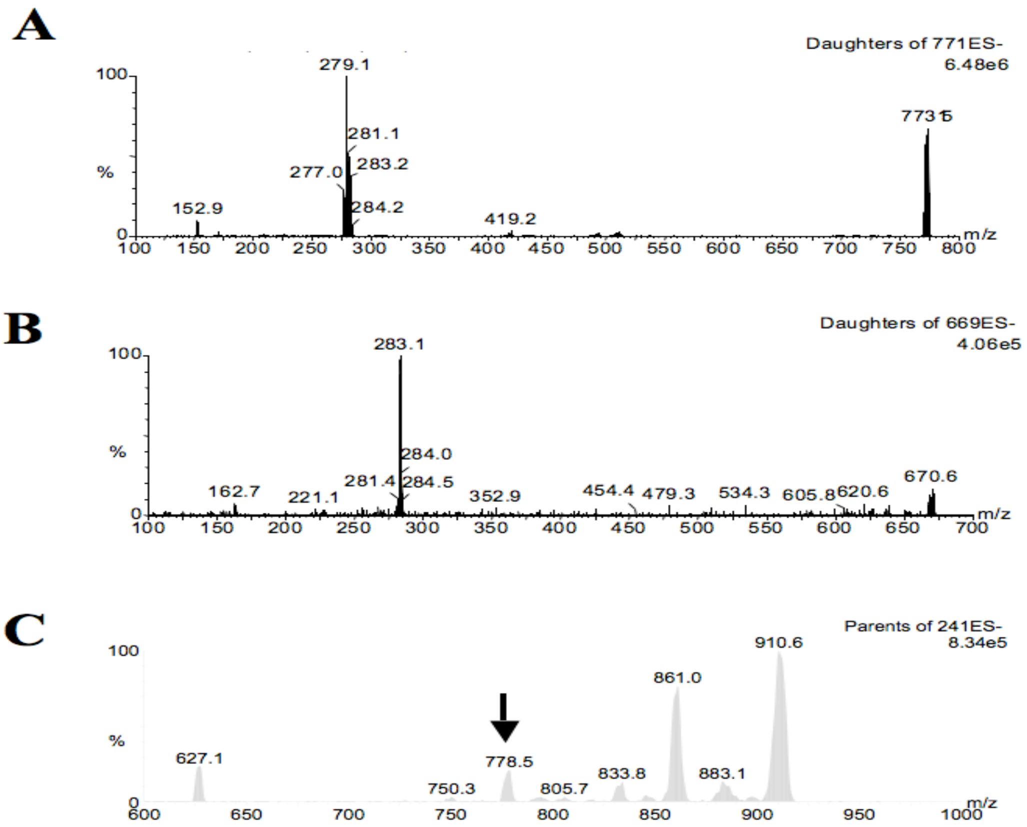

A
Daughters of 771ES-
6.48e6
279.1
281.1
283.2
277.0
284.2
152.9
419.2
%
100
0
m/z
100 150 200 250 300 350 400 450 500 550 600 650 700 750 800
B
Daughters of 669ES-
4.06e5
283.1
284.0
281.4
284.5
162.7
221.1
352.9
454.4
479.3
534.3
605.8
620.6
670.6
%
100
0
m/z
100 150 200 250 300 350 400 450 500 550 600 650 700
C
Parents of 241ES-
8.34e5
910.6
861.0
627.1
778.5
750.3
805.7
833.8
883.1
%
100
0
m/z
600 650 700 750 800 850 900 950 1000

# Figure S8

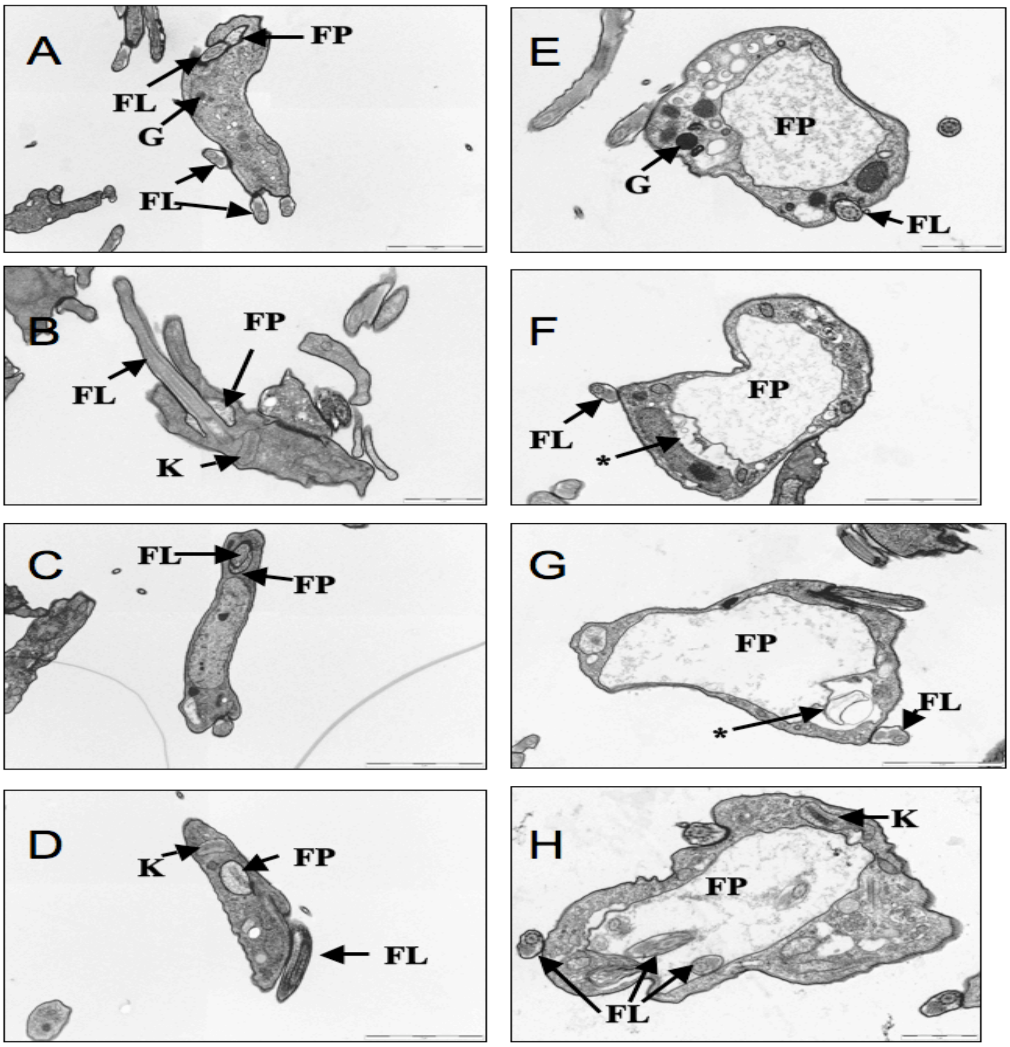

Figure S9

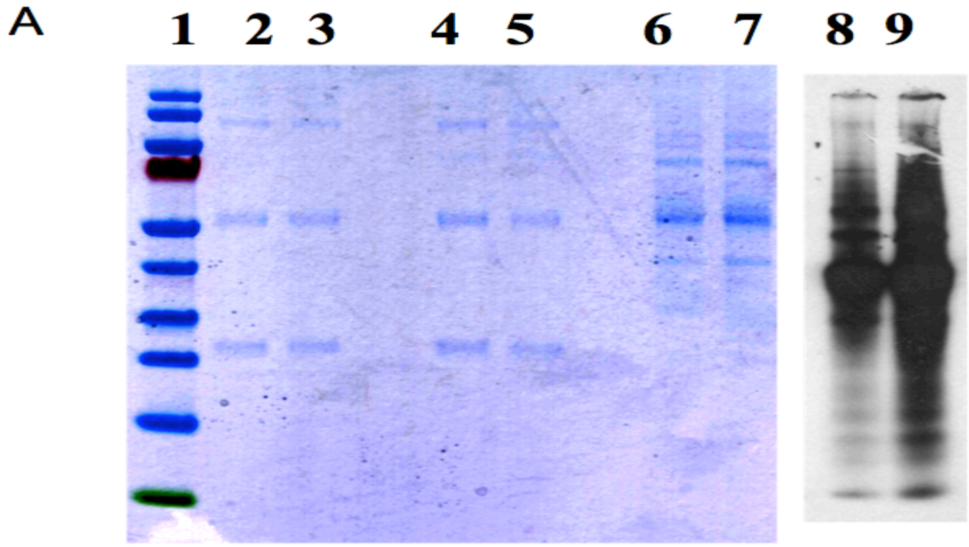


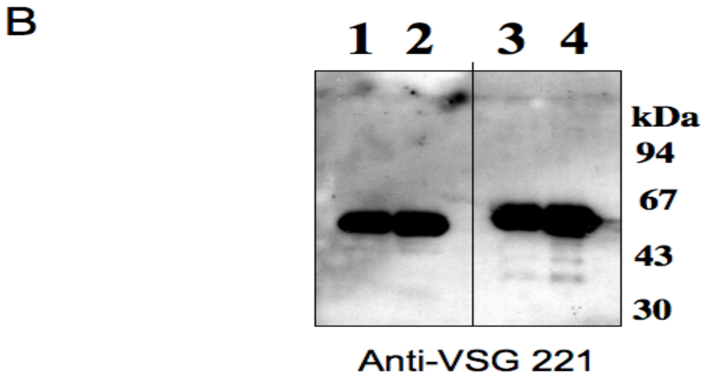

Figure S7

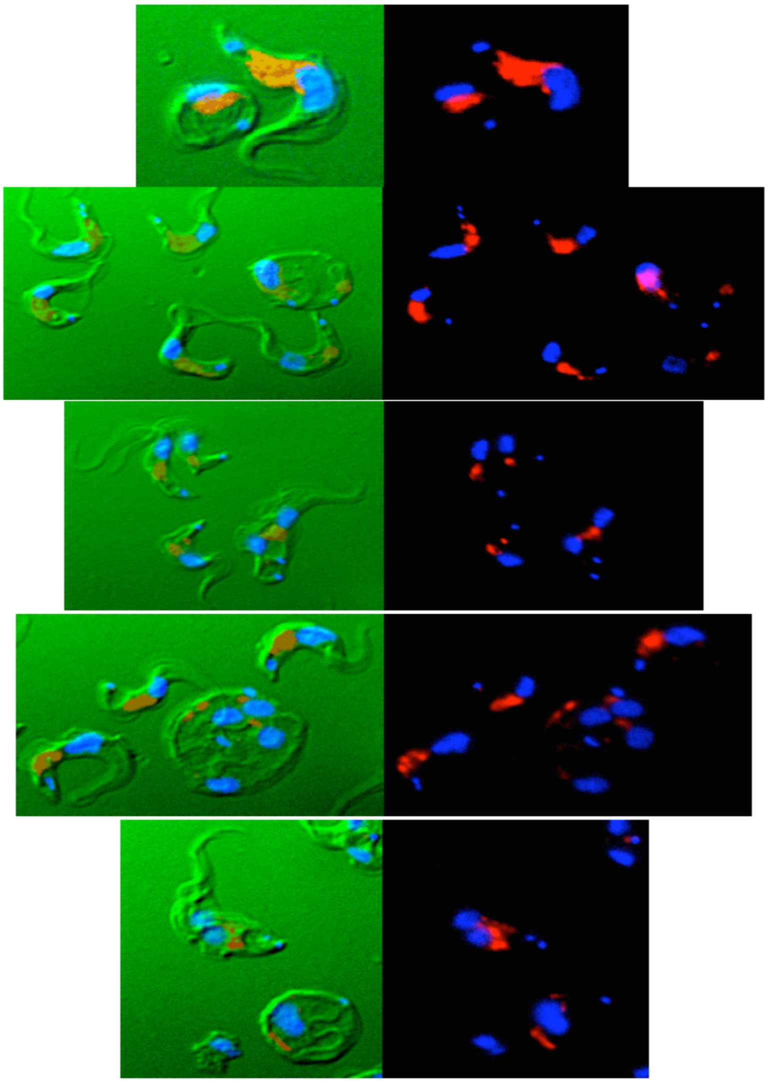

Figure S11

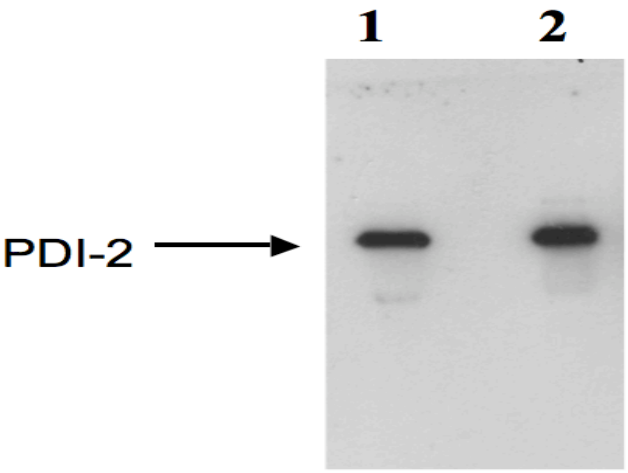

Supplement: Supplementary file 1 [file mmi0076-1461-SD1.pdf]
